# Up-Regulated Expression of LAMP2 and Autophagy Activity during Neuroendocrine Differentiation of Prostate Cancer LNCaP Cells

**DOI:** 10.1371/journal.pone.0162977

**Published:** 2016-09-14

**Authors:** Cecilia Morell, Alicia Bort, Diana Vara-Ciruelos, Ágata Ramos-Torres, Manuel Altamirano-Dimas, Inés Díaz-Laviada, Nieves Rodríguez-Henche

**Affiliations:** 1 Department of Systems Biology, Biochemistry and Molecular Biology Unit, School of Medicine and Health Sciences, University of Alcalá, Alcalá de Henares, Spain; 2 Cell Signalling and Immunology, College of Life Sciences, University of Dundee, Dundee, United Kingdom; 3 Vancouver Prostate Centre. Vancouver, British Columbia V6H 3Z6, Canada; Indian Institute of Science Education and Research, INDIA

## Abstract

Neuroendocrine (NE) prostate cancer (PCa) is a highly aggressive subtype of prostate cancer associated with resistance to androgen ablation therapy. In this study, we used LNCaP prostate cancer cells cultured in a serum-free medium for 6 days as a NE model of prostate cancer. Serum deprivation increased the expression of NE markers such as neuron-specific enolase (NSE) and βIII tubulin (βIII tub) and decreased the expression of the androgen receptor protein in LNCaP cells. Using cDNA microarrays, we compared gene expression profiles of NE cells and non-differentiated LNCaP cells. We identified up-regulation of 155 genes, among them LAMP2, a lysosomal membrane protein involved in lysosomal stability and autophagy. We then confirmed up-regulation of LAMP2 in NE cells by qRT-PCR, Western blot and confocal microscopy assays, showing that mRNA up-regulation correlated with increased levels of LAMP2 protein. Subsequently, we determined autophagy activity in NE cells by assessing the protein levels of SQSTM/p62 and LC3 by Western blot and LC3 and Atg5 mRNAs content by qRT-PCR. The decreased levels of SQSTM/p62 was accompanied by an enhanced expression of LC3 and ATG5, suggesting activation of autophagy in NE cells. Blockage of autophagy with 1μM AKT inhibitor IV, or by silencing Beclin 1 and Atg5, prevented NE cell differentiation, as revealed by decreased levels of the NE markers. In addition, AKT inhibitor IV as well as Beclin1 and Atg5 kwockdown attenuated LAMP2 expression in NE cells. On the other hand, LAMP2 knockdown by siRNA led to a marked blockage of autophagy, prevention of NE differentiation and decrease of cell survival. Taken together, these results suggest that LAMP2 overexpression assists NE differentiation of LNCaP cells induced by serum deprivation and facilitates autophagy activity in order to attain the NE phenotype and cell survival. LAMP2 could thus be a potential biomarker and potential target for NE prostate cancer.

## Introduction

Prostate cancer is the second most common cancer in men, with an estimated 1.1 million cases diagnosed worldwide in 2012 (GLOBOCAN 2012) [1]. Prostate cancer represents an important public health problem throughout the world and for developed countries in particular, since almost 70% of the cases (759,000) occur in more developed regions. Prostate tumors initially depend on androgens. Thus, androgen deprivation therapy (ADT) is used to treat advanced prostate and yields transient efficacy. This therapy consists in administrating LHRH agonists or antagonist which prevent the secretion of the pituitary hormone LH which, in turn, reduces the production of androgens by the testicles [2]. In addition, patients can also receive antiandrogen treatment to block the effects of adrenal residual androgens, this strategy has been termed “combined androgen blockage” [3–5]. Unfortunately, ADT has limited and transient efficacy and most patients receiving it progress to a more aggressive form of the disease termed castration-resistant prostate cancer (CRPC) [5, 6]. The mechanism by which resistance occurs has not been completely elucidated and thus represents a major clinical problem. There is evidence of androgen receptor (AR) reactivation despite decreased serum levels of androgens as an adaptive survival response [4].

One of the hallmarks of advanced prostate cancer is the acquisition of a neuroendocrine phenotype. Neuroendocrine differentiation (NED) is recognized as an adaptation response mechanism to hormonal therapy and represents an aggressive variant of prostate cancer [7, 8]. The amount of NED in prostate adenocarcinoma increases with disease progression and its incidence is expected to increase due to the use of new potent androgen signaling inhibitors in clinical practice [9]. Peptides produced by neuroendocrine (NE) cells, such us neuron-specific enolase (NSE) and chromogranin A, have been detected in the serum of advanced and CRPC patients [10–12]. How NE cells contribute to prostate cancer progression is yet unresolved. These cells are non-mitotic but secrete different neuropeptides and growth factors which could contribute to maintain homeostasis of surrounding cell populations [13]. NED is a highly heterogeneous phenomenon that points to poor prognosis [14, 15]. The origin of NE tumor cells has been hypothesized to arise by transdifferentiation from exocrine tumor cells since NE and exocrine tumor cells from radical prostatectomies share identical allelic profiles [16].

*In vitro*, prostate adenocarcinoma cells have the capacity to transdifferentiate to a neuroendocrine phenotype by exposure to stimuli such as dibutyryl-cyclic AMP [17, 18], forskolin and IL-6 [19], VIP [20] or hormone-depleted medium [21]. The LNCaP cell line is widely used as a cellular model to study the biology of NE transdifferentiation. In this study, we have used LNCaP cells to determine the gene expression profile in NED by performing genome-wide transcriptomic analysis. Our results show up-regulated expression of lysosome-associated membrane glycoprotein 2 (LAMP2) in NE-differentiated LNCaP cells.

LAMP2 is a single-span lysosomal membrane protein which maintains lysosomal stability and participates in autophagy [22]. Structurally, it consists of a polypeptide core of approximately 44 kDa with a short cytoplasmic tail (Ct), a transmembrane domain, and a large luminal domain with extensive N-glycosylation and some O-glycosylation (Nt) which forms a nearly continuous coat on the inner surface of the lysosomal membrane, protecting it from lysosomal proteolitic enzyme hydrolysis [23]. Apart from its role in maintaining the structural integrity of the lysosomal membranes, LAMP2 is critical for lysosomal function [24]. Mice lacking LAMP2 accumulate autophagic vacuoles in several tissues [25]. In humans, mutations in the LAMP2 gene cause Danon disease, an X-linked lysosomal storage disorder characterized by accumulation of vacuolar compartments in heart and skeletal muscle, leading to cardiomyopathy and myopathy [26, 27]. Three spliced variants of the LAMP2 gene generated by alternative splicing have been described: LAMP-2A, LAMP-2B and LAMP-2C, which differ in the transmembrane and cytoplasmic domains [28]. LAMP-2A functions as a receptor of chaperone-mediated autophagy (CMA), a lysosomal proteolytic process known to be activated during starvation that removes damaged cellular proteins [29]; LAMP-2B is more abundantly expressed in muscle and brain and its absence is associated with Danon disease development; the LAMP-2C isoform functions as a receptor for RNA and DNA degradation [30, 31].

Macroautophagy (here referred to as autophagy) has emerged as a way to elude cancer therapy and to promote tumor progression [32, 33]. Prostate cancer treatments such as ADT, taxane and kinase inhibitors usually induce autophagy conferring resistance of prostate cancer cells to therapy. Therefore pharmacological inhibition of autophagy in combination with current prostate cancer therapies or chemotherapy drugs has been proposed as an alternative to improve prostate cancer treatments [34]. The aim of this study was to analyze the role of LAMP2 up-regulation in neuroendocrine differentiation of LNCaP cells and its relationship with autophagy.

## Materials and Methods

### Materials

Bafilomycin A_1_ and 3-methyladenine (3-MA) were from Sigma (St. Louis, MO, USA). LY294002 was from Tocris (Bristol, UK) and Akt inhibitor IV was purchased from Calbiochem (Darmstadt, Germany). Primary antibodies used in this study were: monoclonal anti-NSE from DAKO (Glostrup, Denmark), monoclonal anti-LAMP2 from Abcam (Cambridge, UK), anti-p62, anti-pAkt, anti-Atg5 and anti-GAPDH from Cell Signalling Technology (Danvers, MA, USA), anti-Beclin 1 was from ThermoFisher Scientific (Alcobendas, Spain), polyclonal anti-LC3 from Novus (Abingdon, England, UK) and polyclonal anti-βIII tubulin from Covance (Princeton, NJ, USA). The secondary antibodies were: peroxidase labeled anti-mouse IgG from Sigma (St. Louis, MO, USA) and anti-rabbit IgG from Calbiochem (Darmstadt, Germany).

### Cell culture

The human prostate carcinoma cell line LNCaP was purchased from American Type Culture Collection (ATCC CRL-1740) (Rockville, MD, USA). Cells were used at passages 3 to 20 and routinely grown in complete medium consisting in RPMI 1640 medium containing phenol red and supplemented with 100 IU/ml penicillin G sodium, 100 μg/ml streptomycin sulfate, 0.25 μg/ml amphotericin B (Invitrogen, Paisley, UK) and 10% foetal calf serum. To elicit neuroendocrine differentiation, LNCaP cells were cultured as follows: cells were seeded at a density of 10,000 cells/cm^2^ in complete medium. After 48 hours, the medium was replaced by serum-free medium (SF) and the cells were continuously cultured for 2 to 6 days without splitting. Cells seeded at the same density and cultured at the same time in a complete medium were used as a control. Most of the experiments were carried out with LNCaP cells cultured for 6 days in complete medium (C cells) or in SF medium (NE cells).

### DNA microarray and differential gene expression analysis

Genome-wide transcriptomic analysis was performed using the whole human genome oligo microarray from Agilent platform GPL4133. Total RNA was isolated from LNCaP cells cultured in serum-free medium for 4 hours or 6 days using Trizol reagent according to the manufacturer’s recommended protocol. RNA labeling, hybridization and washing were carried out following Agilent's instructions. Images of hybridized microarrays were acquired with a DNA microarray scanner. Data were background corrected and normalized using the quantile method (Bolstadt et al., 2003). Differential expression analysis was assessed using the linear modeling features of the Limma package from Bioconductor open source software (http://www.bioconductor.org/. DNA microarray assays and bioinformatic data analyses were carried out in the Genomics Unit of the Spanish National Center for Cardiovascular Research (CNIC, Madrid, Spain). Results from differential expression analysis were further analyzed for functional enrichment by using GSEA (Gene Set Enrichment Analysis) v2.2.2 software [35] an open source tool from the Broad Institute (http://software.broadinstitute.org/gsea/index.jsp). Gene Ontology Molecular Process, Cellular Components and Molecular Function gene sets (GO:MP, GO:CC and GO:MF) were downloaded from the Broad Institute’s Molecular Signature Database (version 4.0).

### Western blotting

After treatments, cells were lysed in a lysis buffer (50 mM Tris pH 7.4, 0.8 M NaCl, 5 mM MgCl_2_, 0.1% Triton X-100) containing protease inhibitor and phosphatase inhibitor cocktails (Roche Diagnostics; Mannheim, Germany) and then cleared by microcentrifugation. Total protein content was measured by the BioRad^TM^ protein assay kit (Bio-Rad Laboratories, Richmond, CA, USA) and 20 μg of protein were separated by electrophoresis on 15%–8% sodium dodecyl sulfate polyacrylamide gels. Proteins were transferred onto an Immobilon PVDF membrane (Bio-Rad Laboratories, Richmond, CA, USA) at 100 V for 2 hours at 4°C. Membranes were incubated with the indicated primary antibodies overnight at 4°C and then with the respective secondary antibody. Visualization was performed by incubating the membrane for 3 min with enhanced chemo luminescence detection buffer (100 mM Tris-HCl pH 8.5, 1.25 mM luminol, 0.2 mM p-coumaric acid, and 0.03% H_2_O_2_ and exposed to an Curix RP2 Plus X-ray film (AGFA, Mortsel, Belgium). Densitometric analysis of the blot bands was performed by using Scion Image software (Scion Corporation, Informer Technologies, Inc). GAPDH was used as an internal control. For each blot, fold change in a protein expression level is calculated by dividing the density of each experimental condition by that from control sample. For each protein, data are expressed as the mean of the fold change and standard deviation obtained from at least three independent experiments.

### Confocal microscopy

Cells were grown on glass coverslips and at the end of incubation period were fixed in 4% paraformaldehyde in PBS and incubated with 0.1% Triton X-100 for permeabilization. Immunolabeling with primary antibody was performed by incubation at room temperature for 1h. Secondary labeling was performed with Alexa Flour 594, conjugated to anti-rabbit IgG and Alexa Flour 488 (Invitrogen). Coverslips were then mounted on slides with DAPI-containing mounting medium. Lysosomes were stained by loading cells with 50 nM lysotracker red (Life Technologies, Thermo Fisher Scientific, Waltham, MA USA) for 1 h before the end of the experiment. The cells were then fixed and permeabilized as described above. Imaging was performed with a Leica TCS SP5 laser-scanning confocal microscope with LAS-AF imaging software, using a 63X oil objective. Quantification of the confocal images was performed by using ImageJ v1.46 software (NIH Image) with co-localization analysis plugins for the quantitative co-localization study (Wright Cell Imaging Facility).

### RT-qPCR

RNA extraction was carried out using the Trizol reagent (Invitrogen, Thermo Fisher Scientific, Waltham, MA, USA) according to the manufacturer’s recommended protocol. Two μg of total RNA were reverse transcribed with Transcriptor Reverse Transcriptase (Roche Applied Science, Mannheim, Germany). Real-time quantitative PCR was performed to amplify between 25 and 100 ng of cDNA using the following pairs of primers: human NSE sense 5´-GGCTACACGGAAAAGATCGTTATT-3´ and antisense 5´-GAAGGATCAGTGGGAGACTTGAA-3´; human LAMP2 sense 5´-TGCTGGCTACCATGGGGCTG-3´ and antisense 5´-GCAGCTGCCTGTGGAGTGAGT-3´; ATG sense5´-CAACTTGTTTCACGCTATATCAGG-3´ and antisense 5´-CACTTTGTCAGTTACCAA CGTCA-3´, human LC3 sense5´-TGTCCGACTTATTCGAGAGCAGCA-3´ and antisense 5´-TTCACCAACAGGAAGAAGGCCTGA-3´, human 18S sense5´-GTAACCCGTTGAACCCCATT-3´ and antisense 5´-CCATCCAATCGGTAGTAGCG3´ on the7500 FAST Real Time PCR System (Applied Biosystems) with SYBR Green (Applied Biosystems, Foster City, USA). Target gene expression was normalized to 18S levels in respective samples as an internal control.

### siRNA

Cells were transfected in Opti-MEM® I Reduced Serum Medium (Gibco, Thermo Fisher Scientific) containing 4 μg Lipofectamine®RNAiMAX (Invitrogen, ThermoFisher Scientific) with 100 nM of specific siRNA or control scrambled duplex for 12 h according to the manufacturer’s protocols. The sequences of siRNAs used in this study were: LAMP2 5´-GCUGUGCGGUCUUAUGCAUdTdT-3’ and ATG5: 5’-GUGAGAUAUGGUUUGAAUAdTdT-3’ (Invitrogen, ThermoFisher Scientific, Alcobendas, Spain). For Beclin1 specific Silent Select siRNA was used (Ambion, ThermoFisher Scientific, Alcobendas, Spain). Then the medium was removed and replaced by RPMI containing 10% FBS (control) or serum-free medium (NE) and cultured for 72 h. Afterwards, the cells were transfected a second time using the same conditions and maintained in culture for an additional 72 h. At the end of the incubation, the cells were used for western blot or cell viability assays.

### Cell viability assay

For cell viability assays cells were seeded in a 12-well plate at a 50,000 cells per well and maintained for 48 h. Then the medium was changed for another medium without antibiotic and with or without 10% fetal bovine serum, cells were transfected with the corresponding siRNA and maintained in culture for 6 days. Cell viability was assayed by colorimetric MTT assay.

### Statistical Analysis

All statistical analyze of the microarray data was carried out using R (Smyth GH 2005) in the Genomics Unit of the Spanish National Center for Cardiovascular Research (CNIC, Madrid, Spain). For the rest of the analyses, data are presented as the mean ± S.D. of at least three separate experiments. Statistical significance between groups was tested by Student’s two-tailed and paired t-test using Instat software (Graphad SofwareStat Software, San Diego, CA, USA). Differences among groups were considered significant when P<0.05 or P<0.01 as indicated.

## Results

### Serum deprivation induces neuroendocrine differentiation of LNCaP cells

**Fig 1:**
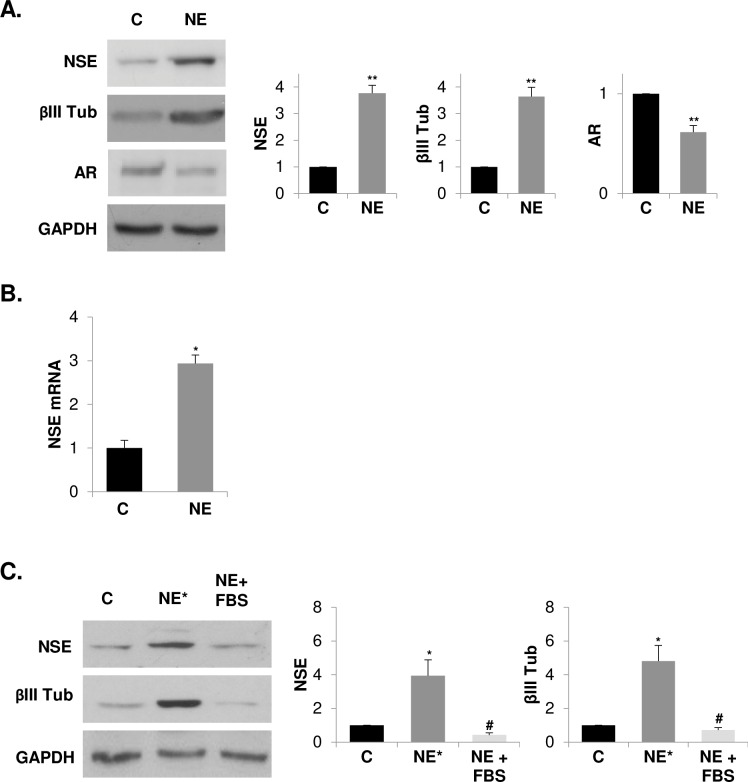
Serum withdrawal induces reversible neuroendocrine differentiation of LNCaP cells. **(A)** LNCaP cells cultured in serum-containing medium (C, control cells) or in serum-free medium (NE, neuroendocrine cells) during 6 days were lysed and neuron-specific enolase (NSE), βIII tubulin (βIII Tub) and androgen receptor (AR) were measured in whole lysates by western blot. GAPDH was used as a loading control. Densitometric analysis of the Western blot bands are shown on the right. **(B)** Quantification of neuron-specific enolase (NSE) mRNA in control and NE cells by real time qRT-PCR. **(C)** LNCaP cells were grown in serum-containing medium (C, control cells), serum-free medium (NE* cells) during 12 days or serum-free medium for the first 6 days and then the medium was replaced by a serum-containing medium and cells were cultured for another 6 days (NE+FBS). Thereafter, cells were lysed and neuron-specific enolase (NSE) and βIII tubulin (βIII Tub) were measured in whole lysates by Western blot. GAPDH was used as a loading control. Densitometric analysis of the Western blot bands are shown on the right. Results are the mean ± S.D. of at least three independent experiments (* p<0.05 and ** p<0.01 versus control cells and # p<0.05 versus neuroendocrine cells, compared by the Student’s t test).

LNCaP cells underwent neuroendocrine (NE) differentiation by serum deprivation of culture medium for 6 days (hereafter called NE cells). βIII Tubulin (βIII Tub) and neuron-specific enolase (NSE) levels were assessed as NE markers. Serum depletion increased the protein levels of βIII Tub and NSE in LNCaP cells in a time-dependent manner (S1 Fig), reaching maximum values at 6 days of serum deprivation. Therefore, LNCaP cells cultured in serum-free medium for 6 days were chosen for the rest of the studies as a NE differentiation model. In accordance with previous reports showing a reduced expression and /or activity of AR [36], NE cells showed a decreased AR expression besides to increased NE markers (Fig 1A). We then assessed the levels of NSE mRNA in NE and control cells by qRT-PCR (Fig 1B) and verified that increased protein levels of NSE correlated with enhanced levels of NSE mRNA in NE cells. In addition, and according to previously reports [18], we corroborated that the neuroendocrine phenotype of LNCaP cells is reversible since the levels of NE markers rise when SF medium is replaced with a complete medium and cells are maintained in culture for an additional 6 days (Fig 1C).

### Differential gene expression in neuroendocrine differentiated LNCaP cells

**Fig 2:**
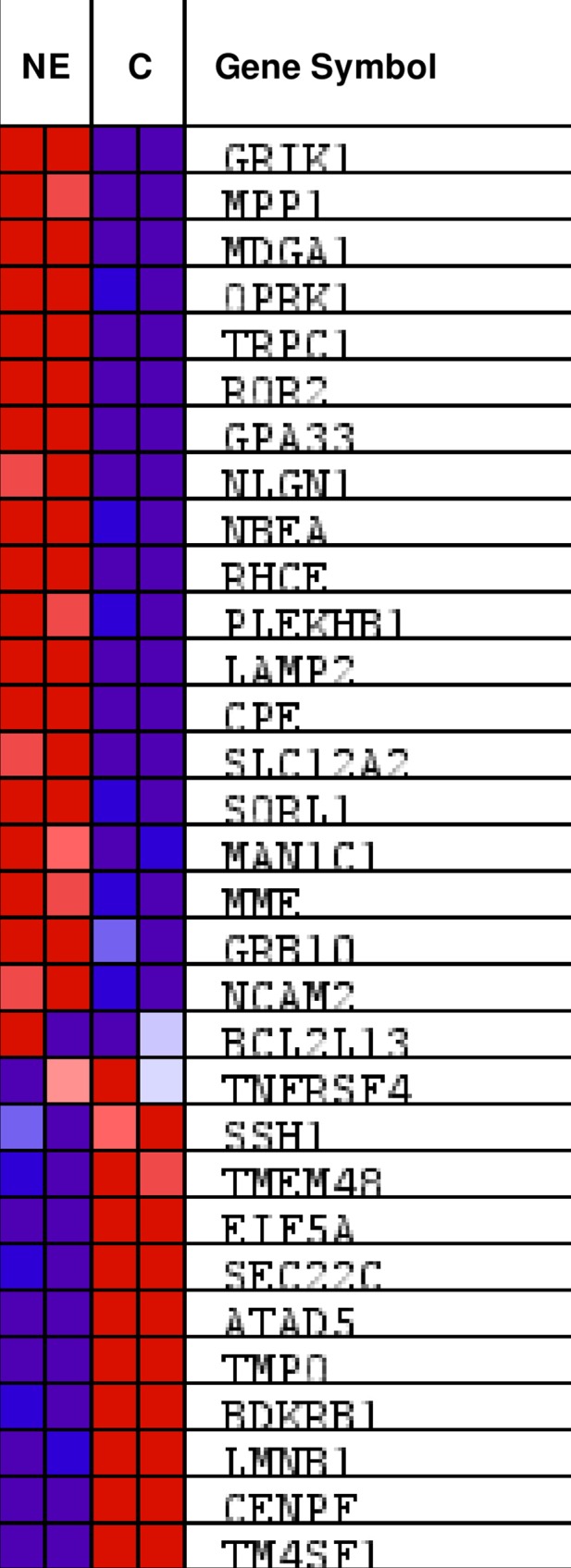
Heat map showing enrichment in membrane components from DEGs between NE and control cells. DEGs between NE and control cells were functionally assessed using GSEA. Gene Ontology gene sets were downloaded from the Broad Institute’s Molecular Signature Database (version 4.0). Enrichment on gene set “intrinsic _to_membrane” is shown. Up-regulated (red) and down-regulated (blue) genes in NE and control cells are shown.

In order to determine the gene expression profile of NE cells and the differentially expressed genes (DEGs) in NE cells versus control cells, we used cDNA microarray technology. From 15,450 genes analyzed in the microarray, 404 of them were differentially expressed in NE cells versus control cells. Among them, 155 genes were up-regulated and 249 genes were down-regulated in NE cells. The list of DEGs in NE cells is shown in supplemental data (S1 and S2 Tables). The analysis of differentially expressed genes corroborated the up-regulation of the NSE gene in NE cells. The functional analysis of DEGs using GSEA v2.2.2 software identified four categories of Biological Process (GO:BP) enrichment in NE cells. The enriched genes of “Anatomical_Structure_Development” and “Signal_Transduction” are shown in supplemental data (S2 Fig and S3 Table). Signal transduction enrichment was due to 22 DEGs from which interleukin 1β (IL1β) and a glutamate ionotrophic receptor kainate type (GRIK1) were the most over expressed genes in NE cells, and cyclin A2 (CCNA2), topoisomerase 2A (TOP2A) and neuromedin U (NMU) were the most down regulated genes in these cells (S2 Fig and S3 Table). By contrast, the category Molecular Function (GO:MF) did not result in any outcome; whereas the Cellular Component (GO:CC) category identified an enrichment in membrane components (“Intrinsic_To_Membrane” and “Integral_To_Membrane”) (Fig 2 and Table 1). Membrane component enrichment was due to 31 DEGs genes (Fig 2), from which 19 genes were up-regulated in NE cells (Table 1). Among the up-regulated genes, we detected high expression of the lysosomal-associated membrane protein 2 (LAMP2), a membrane glycoprotein specific for late endosomes and lysosomes [37] which has been reported to be critical for lysosomal stability and lysosomal degradation of autophagic vacuoles [22, 38].

**Table 1:** List of genes included in the gene set "Intrinsic_to_membrane" that are UP- or DOWN-regulated in neuroendocrine cells. FC*: Fold change.

Symbol	Gene ID	Gene NAME	FC*
CPE	1363	carboxypeptidase E	3,97
GPA33	10223	glycoprotein A33 (transmembrane)	3,42
GRB10	2887	growth factor receptor-bound protein 10	4,12
GRIK1	2897	glutamate receptor, ionotropic, kainate 1	13,86
LAMP2	3920	lysosomal-associated membrane protein 2	3,31
MAN1C1	57134	mannosidase, alpha, class 1C, member 1	3,06
MDGA1	266727	MAM domain containing glycosylphosphatidylinositol anchor 1	4,72
MME	4311	membrane metallo-endopeptidase	3,61
MPP1	4354	membrane protein, palmitoylated 1, 55kDa	5,31
NBEA	26960	neurobeachin	3,82
NCAM2	4685	neural cell adhesion molecule 2	3,18
NLGN1	22871	neuroligin 1	3,58
OPRK1	4986	opioid receptor, kappa 1	9,90
PLEKHB1	58473	pleckstrin homology domain containing, family B (evectins) member 1	3,74
RHCE	6006	Rh blood group, CcEe antigens	3,01
ROR2	4920	receptor tyrosine kinase-like orphan receptor 2	3,74
SLC12A2	6558	solute carrier family 12 (sodium/potassium/chloride transporters), member 2	3,24
SORL1	6653	sortilin-related receptor, L(DLR class) A repeats-containing	3,60
TRPC1	7220	transient receptor potential cation channel, subfamily C, member 1	3,33
ATAD5	79915	ATPase family, AAA domain containing 5	- 2,68
BCL2L13	23786	BCL2-like 13 (apoptosis facilitator)	- 2,66
BDKRB1	623	bradykinin receptor B1	- 3,87
CENPE	1062	centromere protein E, 312kDa	- 15,75
EIF5A	1984	eukaryotic translation initiation factor 5A	- 3,18
LMNB1	4001	lamin B1	- 8,29
SEC22C	9117	SEC22 vesicle trafficking protein homolog C (S. cerevisiae)	- 2,80
SSH1	54434	slingshot homolog 1 (Drosophila)	- 3,18
TM4SF1	4071	transmembrane 4 L six family member 1	- 15,42
TMEM48	55706	transmembrane protein 48	- 5,63
TMPO	7112	thymopoietin	- 3,99

### Confirmation of LAMP2 up regulation in neuroendocrine differentiated LNCaP cells

**Fig 3:**
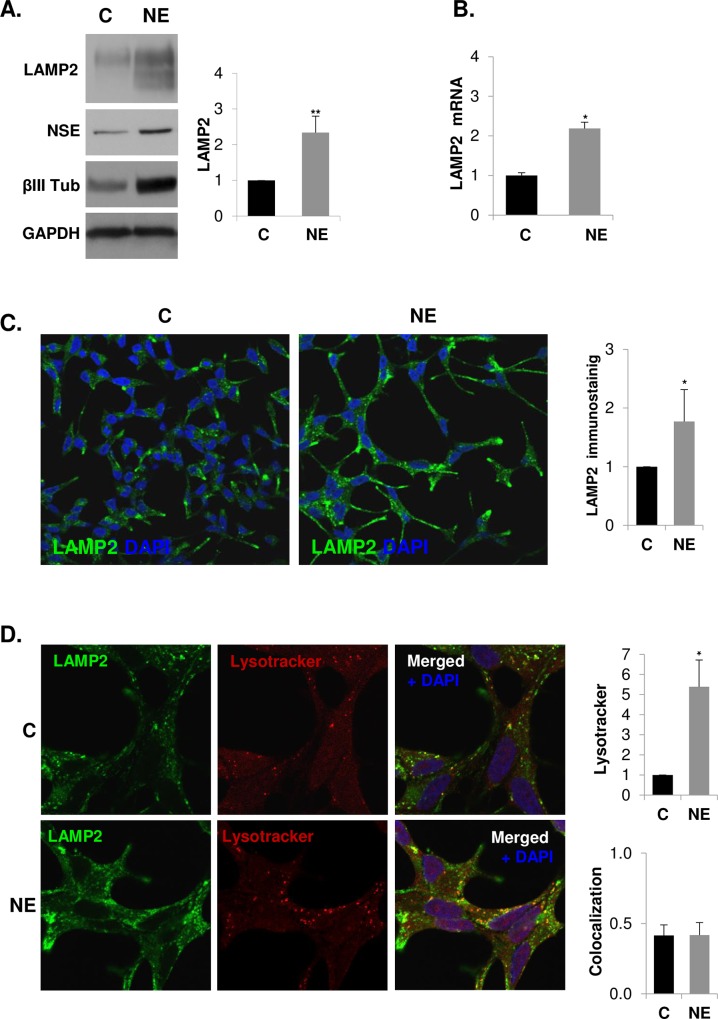
Lysosomal-associated membrane protein 2 (LAMP2) is over-expressed in neuroendocrine differentiated LNCaP cells. LNCaP cells were grown in serum-containing medium (C, control cells) or serum-free medium (NE, neuroendocrine cells) during 6 days. **(A)** LAMP2 was measured in whole lysates of C and NE cells by western blot along with neuroendocrine markers neuron-specific enolase (NSE) and βIII tubulin (βIII Tub). GAPDH was used as a loading control. Densitometric analysis of the Western blot bands are shown on the right. **(B)** Quantification of LAMP2 mRNA in control and NE cells by real time qRT-PCR. Results are the mean ± S.D. of at least three independent experiments (* p<0.05 versus control cells compared by the Student’s t test). **(C)** Detection of LAMP2 by immunofluorescence (green). Nuclei are stained with DAPI (blue). Immunofluorescence was analyzed by confocal microscopy. Quantitative analysis of lysotracker and LAMP2 was performed using ImageJ software (NIH).

To validate the observed LAMP2 up-regulation in NE cells, we assessed LAMP2 protein expression by western blot and mRNA expression by quantitative RT-PCR, showing that NE cells overexpressed both LAMP2 protein and mRNA (Fig 3A and 3B). LAMP2 was observed as a completely glycosylated mature form (∼110 kDa) and less glycosylated form (< 110 kDa). Overexpression of LAMP2 in NE cells was then further evaluated by immunofluorescence analysis. Confocal microscopy and quantitative analysis of confocal images showed a significantly increase of LAMP2 immunoreactivity in NE cells (Fig 3C). LAMP2 accumulated perinuclearly as well as at the edge and at the extensions of NE cells. Since LAMP2 is a membrane protein present in lysosomes, we used lysotracker, an acid-dependent dye, to determine co-localization of LAMP2 and these acidic subcellular compartments (Fig 3D). Lysotracker staining quantification demonstrated increased fluorescence in NE cells suggesting an increase in lysosomes as well as other acidic structures during the NE differentiation process. Moreover, the quantitative analysis of double-fluorescence staining revealed partial co-localization of LAMP2 and such acidic structures. In spite of both LAMP2 and lysotracker fluorescence are notably increased in NE cells, quantitative analysis of co-localization showed a similar extent of co-localization in control and NE cells. On the other hand, LAMP2 expression in plasma membranes has been detected in blood and tumor cells [39, 40] and we cannot rule out increased expression of LAMP2 in NE cells plasma membrane.

### Autophagy is activated in neuroendocrine differentiated LNCaP cells

**Fig 4:**
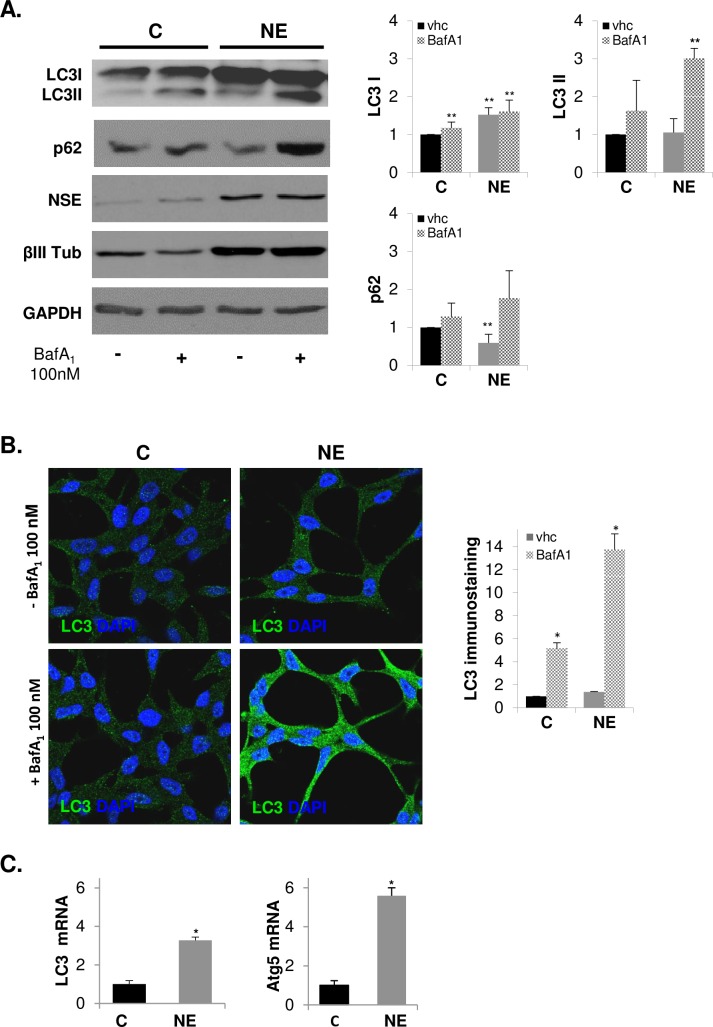
Autophagy is activated in neuroendocrine differentiated LNCaP cells. LNCaP cells were grown in serum containing medium (C, control cells) or serum-free medium (NE, neuroendocrine cells) during 6 days, and when indicated 100 nM Bafilomycin A_1_ was added to the culture medium for the last three hours. **(A)** Autophagy activation markers, LC3 and p62, along with neuroendocrine markers NSE and βIII Tub were measured in whole lysates of C and NE cells by Western blot. GAPDH was used as a loading control. Densitometric analysis of the Western blot bands are shown on the right. **(B)** Detection of LC3 protein by immunofluorescence (green) and nuclei with DAPI (blue). Immunofluorescence was analyzed by confocal microscopy. Quantitative analysis of LC3 was performed using ImageJ software (NIH). **(C)** Quantification of LC3 and Atg5 mRNA in control and NE cells by real time qRT-PCR. Results are the mean ± S.D. of at least three independent experiments (* p<0.05 and ** p<0.01 versus control cells and # p<0.05 versus neuroendocrine cells, compared by the Student’s t test).

The elevated expression of LAMP2 in NE cells along with the previous knowledge of the critical role of LAMP2 for the fusion of autophagic vacuoles with lysosomes [25] suggested that neuroendocrine differentiation might activate autophagy in LNCaP cells. To explore the status of autophagy in NE cells, we determined the protein levels of microtubule-associated protein 1 light chain 3 (LC3) by western blot. During autophagy, the LC3 precursor is cleaved at its C-terminus to form a truncated cytosolic form named LC3-I. LC3-I is then conjugated with phosphatidylethanolamine and bound to the autophagosome membrane to form LC3-II. Therefore, LC3-II levels could be used as an autophagosome accumulation marker. However, as autophagy is a dynamic process, increased levels of LC3-II could also reflect a reduction in autophagosome turnover or even induction of autophagy with impaired turnover ability. To distinguish such situations, LC3-II should be detected in the absence and in the presence of inhibitor of lysosome activity such as lysosomal proteases E64 and pepstatin A or bafilomycin A_1_, which prevents LC3-II turnover by inhibiting lysosomal proteolitic activity and, in the case of bafilomycin A_1_, autophagosome-lysosome fusion [41]. Autophagosome turnover can also be determined by measurement of the adaptor protein sequestosome 1 (SQSTM1/p62), a protein that recruits ubiquitinated proteins to autophagosomes and whose levels inversely correlate with activation of autophagy [41, 42]. NE cells showed an increase in LC3-II and a decrease in p62 levels compared with control LNCaP cells, and such variations were detected when E64 and pepstatin A or 100 nM bafilomycin A_1_ was present in the culture medium during the last three hours (S3 Fig and Fig 4A), suggesting an induction of autophagic flux in NE cells.

To corroborate autophagy activation in NE cells, we labeled endogenous LC3 by immunofluorescence (Fig 4B). Confocal microscopy images showed diffuse cytoplasmic staining of LC3 in control cells and a punctuate LC3 pattern in NE cells, and as expected such switch was notably stronger when bafilomycin A_1_ was present. Quantitative analysis of confocal images revealed a significant increase of LC3 immunostaining in NE cells corroborating western blotting results. It should be note that bafilomycin A_1_ treatment exerted a significantly lower accumulation of LC3 immunostaining in control than in NE cells, suggesting that autophagic flux is greater in NE cells than in control cells.

Although the transcriptional regulation of autophagy has not been closely correlated with functional autophagy, new studies propose a post-transcriptional regulation at the level of gene expression, particularly in long-term autophagic response and in genes involved in the later stages of autophagy [43]. Using qRT-PCR, we determined the mRNA levels of the ATG5 and LC3 genes, which are required for phagophore expansion and autophagosome formation, respectively (Fig 4C). We detected enhanced levels of ATG5 and LC3 mRNA suggesting up-regulated expression of both genes in NE cells. In the case of LC3, increased mRNA levels correlate with greater protein content, suggesting that transcriptional induction may be necessary to replenish the LC3 protein that is turned over during autophagy flux activation.

### Autophagy inhibition prevents neuroendocrine differentiation of LNCaP cells

**Fig 5:**
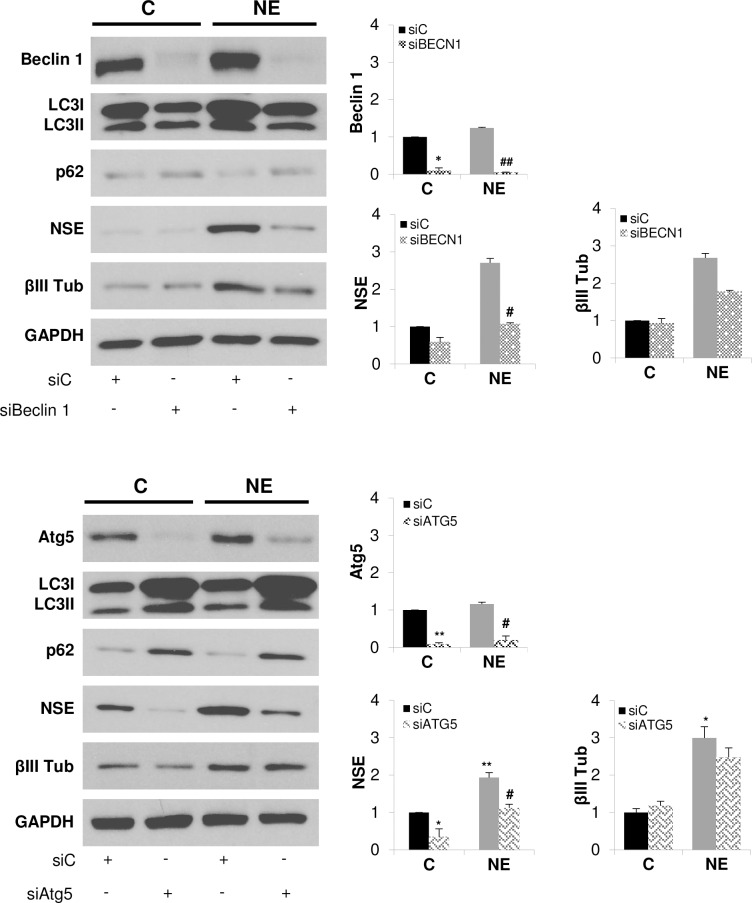
Autophagy blocking prevents neuroendocrine differentiation of LNCaP cells. LNCaP cells grown in serum-containing medium (C, control cells) or serum-free medium (NE, neuroendocrine cells) were transfected with negative control siRNA (siC) or siRNA targeting Beclin1 (siBeclin1) or siRNA targeting Atg5 (siATG5) during 6 days. Thereafter, cells were lysed and Beclin1 or Atg5, autophagy markers, LC3 and p62, and neuroendocrine markers, NSE and βIII Tub were measured in whole lysates by Western blot. GAPDH was used as a loading control. Densitometric analysis of the Western blot bands are shown on the right. Results are the mean ± S.D. of at least three independent experiments (*p<0.05 and **p<0.01 versus non-treated control cells and # p<0.05 versus non-treated neuroendocrine cells, compared by the Student’s t test).

To assess the role of autophagy activity on neuroendocrine differentiation of LNCaP cells, we silenced Beclin1, an autophagy gene which interacts with class III PI3K to initiate autophagosome formation, and Atg5, which form part of the complex Atg5-Atg12/Atg16L involved in LC3 lipidation (Fig 5). Loss of expression of Beclin 1 and Atg5 was verified by Western blotting. Next, we evaluated autophagy activity by measuring LC3 and p62 protein levels. Beclin1 and Atg5 silencing resulted in accumulation of p62 protein in both control and NE cells, demonstrating an efficient blockage of autophagy. In the case of LC3 levels, we observed that Beclin1 and Atg5 knockdown exerted opposite effect, while Beclin1 knockdown decreased LC3I protein levels, Atg5 elicited notably accumulation of LC3I in both control and NE cells. Regarding NE markers, Beclin 1 and Atg5 knockdown significantly reduced NSE protein levels in NE cells and, in a less extent, those of βIII tub. Taken together, those results clearly demonstrate that autophagy activity supports NED of LNCaP cells.

### LAMP2 knockdown prevents autophagy, neuroendocrine differentiation and survival of LNCaP cells

**Fig 6:**
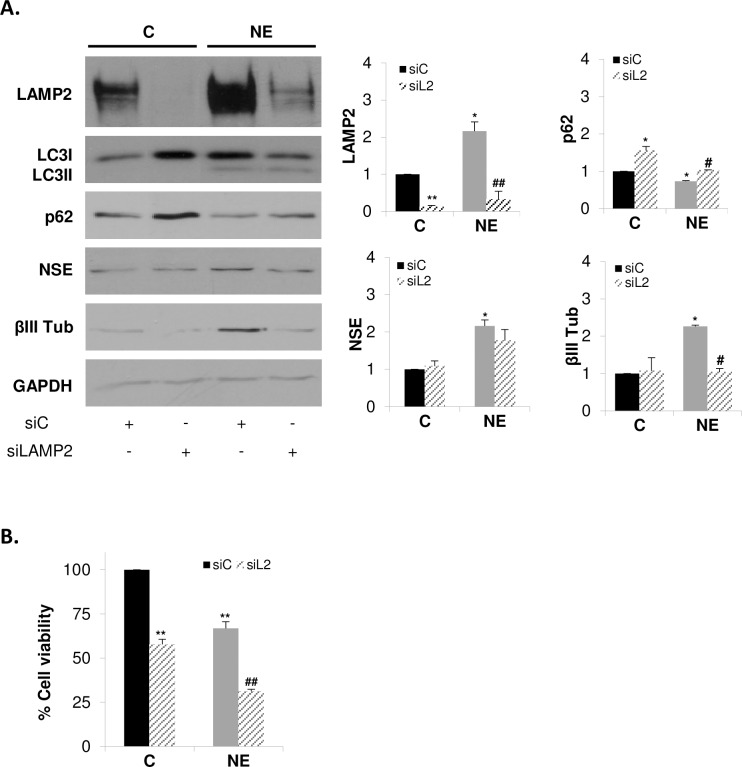
LAMP2 knockdown prevents autophagy, neuroendocrine differentiation and survival of LNCaP cells. LNCaP cells were transfected with negative control siRNA (siC) or siRNA targeting LAMP2 (siLAMP2) and grown in serum-containing medium (C, control cells) or serum-free medium (NE, neuroendocrine cells) during 6 days. **(A)** Cells were lysed and LAMP2, autophagy markers, LC3 and p62, and neuroendocrine markers, NSE and βIII Tub, were measured in whole lysates by Western blot. GAPDH was used as a loading control. Densitometric analysis of the Western blot bands are shown on the right. **(B)** Cell viability of control and NE cells treated with siC or siLAMP2 was monitored by MTT assay. Results are the mean ± S.D. of at least three independent experiments (* p<0.05 and ** p<0.01 versus siC transfected control cells and # p<0.05 and ## p<0.01 versus siC transfected neuroendocrine cells, compared by the Student’s t test).

To answer the question of whether LAMP2 up-regulation is required to induce NED and autophagy in LNCaP cells, LAMP2 was knocked down in control and NE cells by interference RNA. Loss of expression of LAMP2 in cells transfected with siLAMP2 was verified by Western blotting (Fig 6A). Silencing of LAMP2 in NE cells significantly decreased the levels of the neuroendocrine marker βIII Tub but not those of NSE. Autophagy activity was evaluated by measuring p62 levels in whole lysates. siLAMP2 resulted in an accumulation of the cargo protein p62 in both control and NE cells, indicating that knocking down of LAMP2 blocks the fusion of autophagosomes and lysosomes, as has been previously reported [25]. In addition, LAMP2 knockdown significantly reduced cell viability of both control and NE LNCaP cells (Fig 6B). These findings strongly support a role of LAMP2 on autophagy activity as well as on survival and NED of LNCaP cells.

### Autophagy inhibition decreases LAMP2 levels and survival of neuroendocrine differentiated LNCaP cells

**Fig 7:**
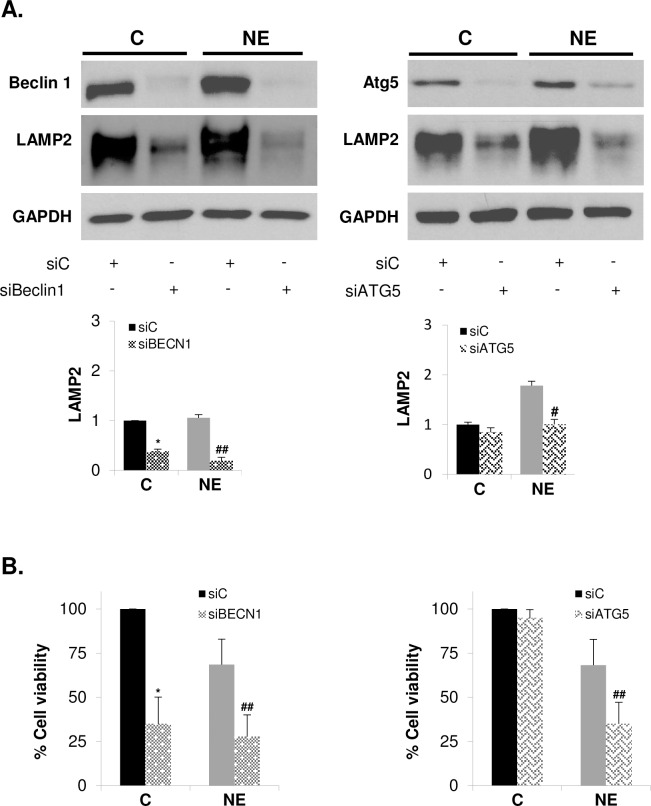
Autophagy inhibition decrease LAMP2 levels and survival of neuroendocrine differentiated LNCaP cells. LNCaP cells were transfected with negative control siRNA (siC) or siRNA targeting Beclin1 (siBeclin1) or si RNA targeting Atg5 (SiATG5) and grown in serum containing medium (C, control cells) or serum-free medium (NE, neuroendocrine cells) during 6 days. **(A)** Cells were lysed and Beclin1 (left), Atg5 (right) and LAMP2 were measured in whole lysates by Western blot. GAPDH was used as a loading control. Densitometric analysis of the Western blot bands are shown below. **(B)** Cell viability of control and NE cells treated with siC, siBeclin1 (left) or SiATG5 (right) was monitored by MTT assay. Results are the mean ± S.D. of three independent experiments (* p<0.05 versus siC transfected control cells and # p<0.05 and ## p<0.01 versus siC transfected neuroendocrine cells, compared by the Student’s t test).

Next, we determine whether autophagy activity play a role in regulating LAMP2 expression. We examine LAMP2 protein levels in Beclin1 and Atg5 silenced control and NE LNCaP cells (Fig 7A). Blockage of autophagy by knocking down Beclin1 or Atg5 significantly decrease LAMP2 protein levels in NE cells. A similar effect was also observed in control LNCaP cells. Since LAMP2 knockdown elicited a dramatic decrease in cell survival, we examine cell viability in Beclin1 and Atg5 silenced cells (Fig 7B). Beclin1 knockdown significantly decreased survival of both control and NE cell, whether such effect is related with diminished levels of LC3 observed in Beclin 1 silenced cells (Fig 5) should be further analyzed. In contrast, Atg5 knockdown had no effect on viability of control LNCaP cells but significantly decreased cell survival of NE cells. These results demonstrated that autophagy activity contributes to regulates LAMP2 protein level and NE cells survival.

### Inhibition of AKT blocks autophagy and prevents overexpression of LAMP2 and neuroendocrine differentiation of LNCaP cells

**Fig 8:**
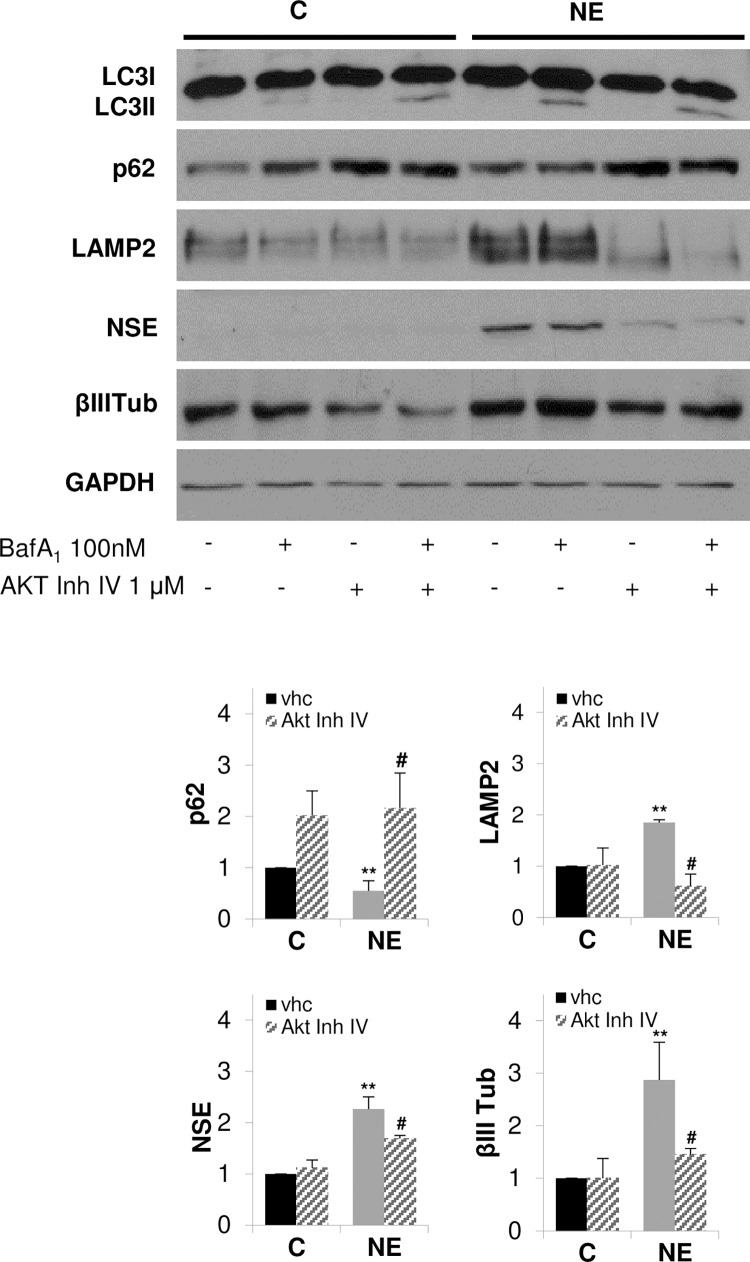
Pharmacological inhibition of AKT prevents autophagy, LAMP2 overexpression and neuroendocrine differentiation of LNCaP cells. LNCaP cells were grown in serum-containing medium (C, control cells) or serum-free medium (NE, neuroendocrine cells) during 6 days. Cells where treated with vehicle (vhc) or 1 μM AKT inhibitor IV for 6 days. When indicated, cells were treated with 100 nM bafilomycin A_1_ for the last three hours in culture. Thereafter cells were lysed and autophagy markers, LC3 and p62, LAMP2 and neuroendocrine markers, NSE and βIII Tub, were measured in whole lysates by Western blot. GAPDH was used as a loading control. Densitometric analysis of the Western blot bands are shown on the right. Results are the mean ± S.D. of at least three independent experiments (* p<0.05 and **p<0.01 versus not treated control cells and # p<0.05 versus not treated neuroendocrine cells, compared by the Student’s t test).

We and others have reported the importance of the PI3K/AKT/mTOR pathway for NE differentiation [20, 44, 45]. In addition, we have very recently demonstrated that serum deprivation of LNCaP cells increases AKT phosphorylation at Ser473 as well as the phosphorylation of its downstream signaling protein S6, which correlates with the increase in NE markers expression [46]. Here, and to gain a deeper understanding of the relationship between LAMP2, autophagy and NE differentiation of LNCaP cells, we investigated the effect of the pharmacological inhibition of AKT on autophagy, LAMP2 and NE markers expression in LNCaP cells (Fig 8). In some conditions, cells were treated with Bafilomycin A_1_ during the last three hours in culture in order to block autophagy flux for better recover LC3II signal. Treatment of cells with the selective AKT inhibitor IV resulted in accumulation of the cargo protein p62, and a decrease of the expression of LAMP2 and NE markers in NE cells, indicating that AKT activity regulates autophagic flux, LAMP2 expression and neuroendocrine differentiation.

## Discussion

NED is one of the hallmarks of advanced castration-resistant prostate cancer and has been proposed as a mechanism of resistance to current therapies [8]. Using expression microarrays, we have identified 404 genes differentially expressed between NE-differentiated and non-differentiated LNCaP cells. Among them, 155 genes were up-regulated including LAMP2, and 249 genes were down-regulated in NE cells.

LAMP2 plays an important role in lysosomal stability as well as in autophagy. Lysosomes are cellular compartments that digest and recycle materials and cancer cells are dependent of effective lysosomal function [47]. A reduction of LAMP2 protein levels triggers lysosomal membrane permeabilization and sensitizes cells to the lysosomal cell death pathway [48]. Several lines of evidence associate enhanced levels of LAMP2 with tumor progression. The majority of cancer cells display LAMP2 expression and, surprisingly, the prostate along with the thyroid gland are the tissues which show the strongest immunoreativity against LAMP2 (the human protein atlas; http://www.proteinatlas.org/). In colorectal cancer epithelium, LAMP1 and LAMP2 levels are increased, suggesting that LAMPs are related to neoplastic progression, but there is no direct association between such up-regulated expression and cell proliferation [49]. Our results show that in NE cells, LAMP2 is up-regulated, both at the level of mRNA. This overexpression was accompanied by increased autophagy activity. In addition, knockdown of LAMP2 hampered NED, as demonstrated by the decreased levels of NE markers, cell survival and prevented autophagy, as demonstrated by p62 protein accumulation. To our knowledge, this is the first report showing the involvement of LAMP2 in NE differentiation of prostate cancer cells. Lysosomes are the terminal degradative compartment for autophagy and LAMP2 plays a critical role in the fusion of autolysosomes with lysosomes to form the hybrid structure called autophagolysosome. Reduced fusion has been shown in cells depleted of LAMP2 as demonstrated in LAMP2 knockout mice which showed accumulation of autophagic vacuoles in several tissues [25] and in patients with Danon disease [26, 27]. The increased expression of LAMP2 could help NE cells increase the fusion activity between lysosomes and autophagosomes, leading to the increased autophagy activity shown in NE cells and constituting a survival signal for NE cells. In breast tumors, LAMP-2A overexpression leads to CMA activation and cancer cell survival [50]. Moreover, Kon *et al*. have been shown overexpression of LAMP-2A and CMA activation in more than 40 different types of human tumors when compared with normal tissue surrounding the tumors. In line with this, blockage of CMA reduces the metastatic capacity of lung cancer cells due to the reduced capacity of tumor cells to sustain their enhanced metabolic activity [51]. However, we have not investigated whether CMA was enhanced in NE cells. This question will be addressed in future studies.

LAMP2 expression is not exclusive of lysosomal and late endosomal membranes. It can also be associated to the cell plasma membrane. Human peripheral blood mononuclear cells (PBMC) have been shown to express LAMP2 in their plasma membrane. Moreover, stimulation of PBMC with the lectin phytohaemagglutinin increased LAMP2 levels, a rise which was involved in PBMC adhesion to vascular endothelium [39]. More recently, Damaghi et al. have shown LAMP2 overexpression in plasma membranes of MCF-7 breast cancer cell line, adapted to grow in acidic conditions, both *in vitro* and *in vivo* [40]. They extended their studies to breast cancer patients and showed enhanced levels of LAMP2 in breast cancer tumors as compared to normal tissue. This increase correlated with increased tumor progression. The authors explain the overexpression of LAMP2 as an adaptation mechanism to chronic acidosis in the tumor microenvironment, since depletion of LAMP2 is sufficient to increase acidosis-mediated toxicity and, interestingly, tap-water bicarbonate sodium therapy reduces LAMP2 expression. They propose the use of LAMP2 as a marker to quantify the presence of acidity in biopsies of solid tumors as well as a novel therapeutic target [40]. It is important to note that in TRAMP mice, an animal model of prostate cancer that displays NE differentiation, systemic sodium bicarbonate buffer administration inhibits carcinogenesis [52]. It will be interesting to investigate the expression levels of LAMP2 in tumors of this prostate cancer animal model to elucidate its possible role in tumor progression, and extend these studies to other NE prostate cancer models such as xenografts.

Macroautophagy (here referred to autophagy) has emerged as a way to elude cancer therapy and to promote tumor progression [32, 33]. Prostate cancer treatments such as ADT, taxane and kinase inhibitors usually induce autophagy conferring resistance of the prostate cancer cells to therapy. Hence, pharmacological inhibition of autophagy in combination with current prostate cancer therapies or chemotherapy drugs may improve prostate cancer treatments [34]. Here, we show significant increase of ATG5 and LC3 mRNA levels as assessed by qRT-PCR that correlates with increased levels of LC3 protein and elevated autophagy flux, as assessed by decreased levels of p62 in NE cells. In addition, the pharmacological and molecular inhibition of autophagy, besides blocking autophagy flux, prevents NE differentiation of LNCaP cells, as shown by decreased levels of NE markers as well as cell survival. These results demonstrate that autophagy activation is required for survival and NE differentiation of LNCaP cells. The importance of PI3K/AKT/mTOR pathway on autophagy regulation is well established [53]. During starvation, mTOR is inhibited allowing autophagy to be activated. Surprisingly, here we observed that autophagy is activated in NE cells along with PI3K/AKT/mTOR activation, maybe because, as has been previously described [54], in prolonged starvation systems mTOR reactivation is required for the degradation of autolysosomes. We cannot discard such possibility in our model of NED since serum-starvation lasts for 6 days. On the other hand, Matsuda-Lennikov et al. have recently demonstrated that accumulation of AKT at the lysosomal membrane is critical for autophagy induction [55]. It seems that spatial control of AKT determines autophagy activity, thus AKT targeted to plasma membrane via class I PI3K production of PtdIns(3,4,5)P_3_ inhibits autophagy, while AKT targeted the lysosome via class III PI3K generation of PtdIns(3)P promotes autophagy [55, 56]. We showed that AKT inhibition disrupts autophagy and NE differentiation of LNCaP cells, however we not known AKT subcellular localization. Accordingly with our results, Fan et al. reported that AKT and autophagy cooperate to promote survival of drug-resistance glioma [57].

Autophagy activation is also reported in NE differentiation induced by IL-6 treatment of LNCaP, conferring resistance to etoposide treatment [58]. IL-6 treatment down-regulates the expression of REST (repressor element-1 silencing transcription factor) [58, 59], a neuronal transcriptional repressor known to be down-regulated in neuroendocrine prostate cancer [60], and the decreased levels of REST allow LNCaP cells to activate autophagy [58]. In our model of NED, we did not observe changes in the expression of REST, suggesting that the driving force to achieve the NE phenotype is quite different in IL-6-treated and serum-starved LNCaP cells. In our model, we have demonstrated that serum-deprivation induction of NE marker expression is correlated with increased AKT activity and decreased AMPK activity and that the cannabinoid WIN 55,212–2 inhibits PI3K/AKT, resulting in AMPK activation and prevention of NED [46]. In this study, we extend these results showing that the induction of autophagy by serum starvation is dependent on AKT activity since AKT inhibitor IV blocks autophagy and prevents overexpression of both LAMP2 and NE markers. A role of autophagy in protein trafficking and secretion pathway has been proposed [61, 62], so the activation of autophagy and LAMP2 overexpression observed in our model of NED could be related with the secretory activity of neuroendocrine cells.

In summary, we describe, for the first time, the up-regulation of LAMP2 as a survival signal for NE differentiation of LNCaP cells induced by serum deprivation during 6 days. LAMP2 could thus be a molecular component of NE differentiation and could be used as a NE marker. Further studies are warranted in order to gain a better understanding of the complex NE differentiation process.

## Supporting Information

S1 Fig Time course variation of NE marker proteins in LNCaP cells cultured in serum free medium. (PPTX)

S2 Fig Heat map showing enrichment in GO:BP “Anatomical_Structure_Development” and “Signal_Transduction” from DEGs between NE and control cells. (PPTX)

S3 Fig Time course of LC3 levels in LNCaP cells cultured in serum-free medium. (PPTX)

S1 Table DEGs in NE cells. Up-regulated genes.
(DOCX)

S2 Table DEGs in NE cells. Down-regulated genes.
(DOCX)

S3 Table List of genes included in GO: BP "Anatomical_Structure_Development" and "Signal_Transduction" gene sets that are UP and DOWN-regulated in neuroendocrine cells. FC*: Fold change.
(DOCX)
