# Supplementary material for: Up-Regulated Expression of LAMP2 and Autophagy Activity during Neuroendocrine Differentiation of Prostate Cancer LNCaP Cells
Source: PLoS One. 2016 Sep 14;11(9):e0162977. doi: 10.1371/journal.pone.0162977 (PMC5023108; doi:10.1371/journal.pone.0162977)
Supplement: S1 Fig — (PPTX) [file pone.0162977.s001.pptx]

## Slide 1
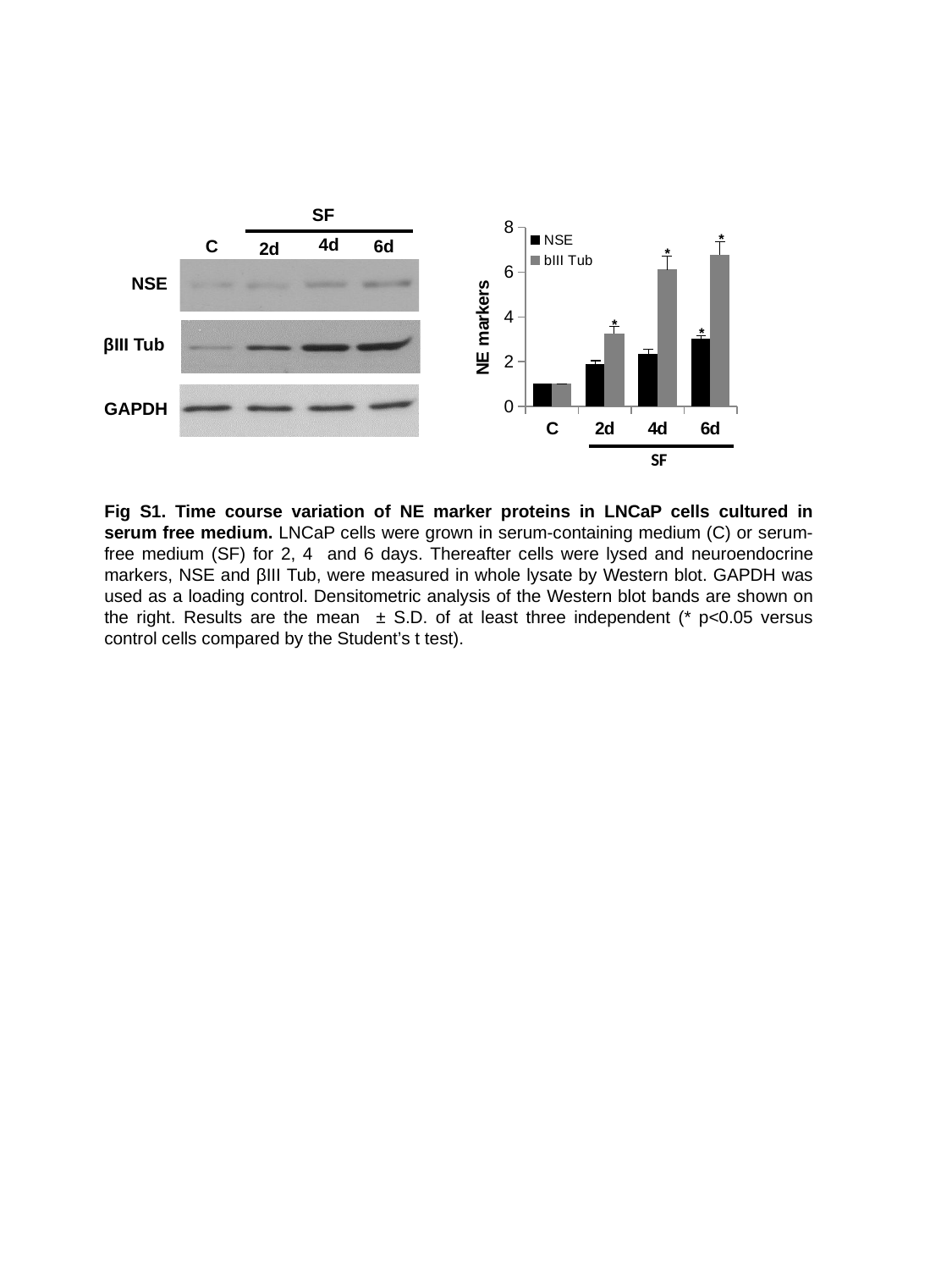

SF
4d
C
6d
2d
NSE
βIII Tub
GAPDH
### Chart
| Category | | |
|---|---|---|
| C | 1.0 | 1.0 |
| 2d | 1.8597106532222698 | 3.240984544934188 |
| 4d | 2.328803156510303 | 6.1053234115627015 |
| 6d | 2.995835160017536 | 6.774184315970235 |SF
*
*
*
*
Fig S1. Time course variation of NE marker proteins in LNCaP cells cultured in serum free medium. LNCaP cells were grown in serum-containing medium (C) or serum-free medium (SF) for 2, 4 and 6 days. Thereafter cells were lysed and neuroendocrine markers, NSE and βIII Tub, were measured in whole lysate by Western blot. GAPDH was used as a loading control. Densitometric analysis of the Western blot bands are shown on the right. Results are the mean ± S.D. of at least three independent (* p<0.05 versus control cells compared by the Student’s t test).
