# Supplementary material for: Up-Regulated Expression of LAMP2 and Autophagy Activity during Neuroendocrine Differentiation of Prostate Cancer LNCaP Cells
Source: PLoS One. 2016 Sep 14;11(9):e0162977. doi: 10.1371/journal.pone.0162977 (PMC5023108; doi:10.1371/journal.pone.0162977)
Supplement: S2 Fig — (PPTX) [file pone.0162977.s002.pptx]

## Slide 1
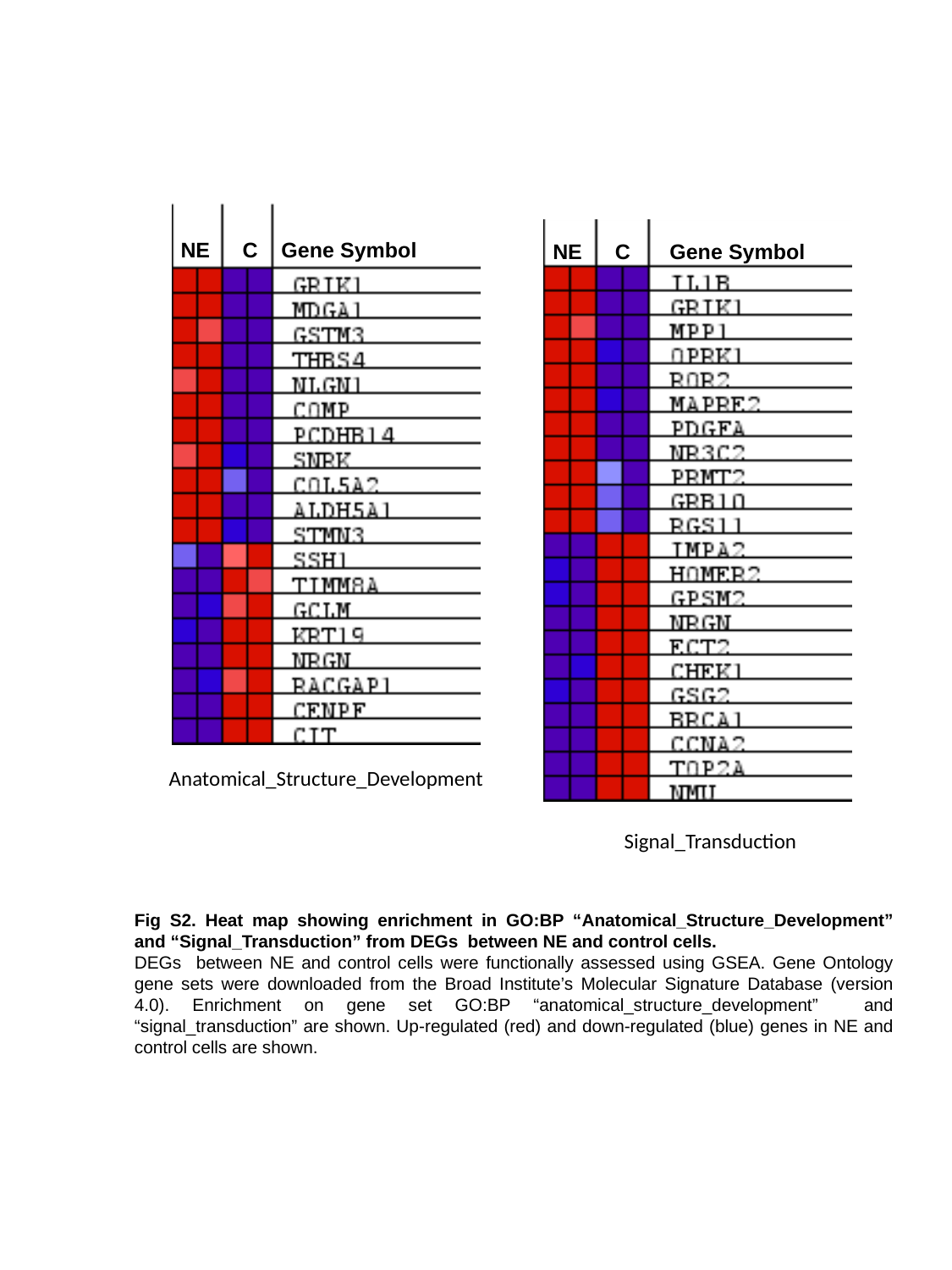

Supplemental2
Enrichment genes expression in NE cells.
NE
C
Gene Symbol
NE
C
Gene Symbol
Anatomical_Structure_Development
Signal_Transduction
Fig S2. Heat map showing enrichment in GO:BP “Anatomical_Structure_Development” and “Signal_Transduction” from DEGs between NE and control cells.
DEGs between NE and control cells were functionally assessed using GSEA. Gene Ontology gene sets were downloaded from the Broad Institute’s Molecular Signature Database (version 4.0). Enrichment on gene set GO:BP “anatomical_structure_development” and “signal_transduction” are shown. Up-regulated (red) and down-regulated (blue) genes in NE and control cells are shown.
