# Supplementary material for: Up-Regulated Expression of LAMP2 and Autophagy Activity during Neuroendocrine Differentiation of Prostate Cancer LNCaP Cells
Source: PLoS One. 2016 Sep 14;11(9):e0162977. doi: 10.1371/journal.pone.0162977 (PMC5023108; doi:10.1371/journal.pone.0162977)
Supplement: S3 Fig — (PPTX) [file pone.0162977.s003.pptx]

## Slide 1
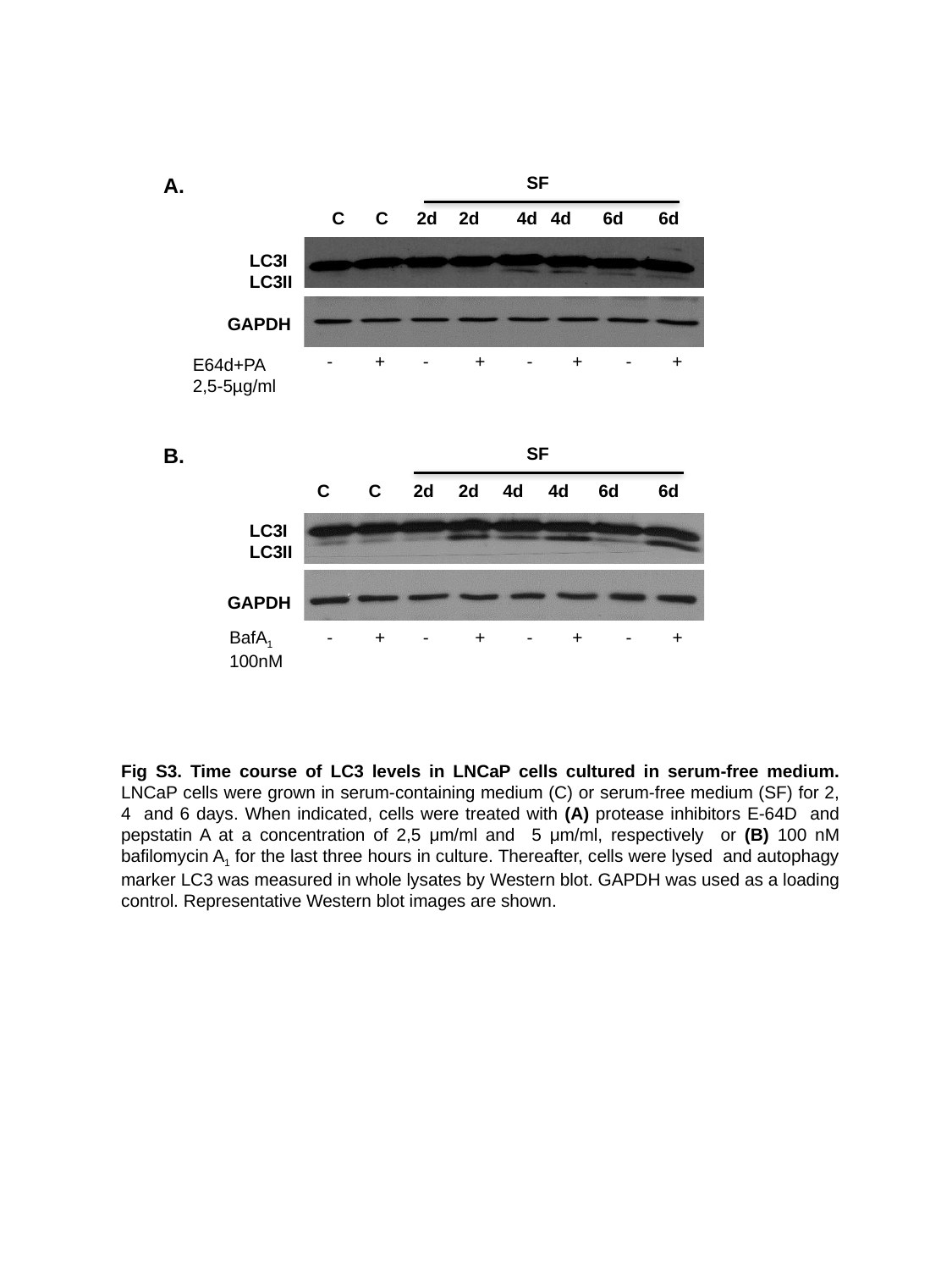

A.
SF
C
C
2d
2d
4d
4d
6d
6d
LC3I
LC3II
GAPDH
-
+
-
+
-
+
-
+
E64d+PA
2,5-5µg/ml
B.
SF
C
C
2d
2d
4d
4d
6d
6d
LC3I
LC3II
GAPDH
-
+
-
+
-
+
-
+
BafA1
100nM
Fig S3. Time course of LC3 levels in LNCaP cells cultured in serum-free medium. LNCaP cells were grown in serum-containing medium (C) or serum-free medium (SF) for 2, 4 and 6 days. When indicated, cells were treated with (A) protease inhibitors E-64D and pepstatin A at a concentration of 2,5 μm/ml and 5 μm/ml, respectively or (B) 100 nM bafilomycin A1 for the last three hours in culture. Thereafter, cells were lysed and autophagy marker LC3 was measured in whole lysates by Western blot. GAPDH was used as a loading control. Representative Western blot images are shown.
