# Supplementary material for: Up-Regulated Expression of LAMP2 and Autophagy Activity during Neuroendocrine Differentiation of Prostate Cancer LNCaP Cells
Source: PLoS One. 2016 Sep 14;11(9):e0162977. doi: 10.1371/journal.pone.0162977 (PMC5023108; doi:10.1371/journal.pone.0162977)
Supplement: S1 Table — Up-regulated genes. (DOCX) [file pone.0162977.s004.docx]

**Table S1: DEGs in NE cells. Up-regulated genes**

Genes up-regulated in neuroendocrine differentiated LNCaP cells induced by serum deprivation

| **SYMBOL** | **GENE ID** | **GENE NAME** | **FC*** |
| --- | --- | --- | --- |
| ABLIM2 | [84448](http://www.ncbi.nlm.nih.gov/entrez/query.fcgi?db=Nucleotide&cmd=search&term=A_23_P255672) | actin binding LIM protein family, member 2 | 2,77 |
| ABP1 | [00026](http://www.ncbi.nlm.nih.gov/entrez/query.fcgi?db=Nucleotide&cmd=search&term=A_23_P59452) | amiloride binding protein 1 (amine oxidase (copper-containing)) | 9,44 |
| ALDH5A1 | [07915](http://www.ncbi.nlm.nih.gov/entrez/query.fcgi?db=Nucleotide&cmd=search&term=A_24_P115007) | aldehyde dehydrogenase 5 family, member A1 (succinate-semialdehyde dehydrogenase) | 2,59 |
| AMT | [00275](http://www.ncbi.nlm.nih.gov/entrez/query.fcgi?db=Nucleotide&cmd=search&term=A_23_P257164) | aminomethyltransferase | 2,94 |
| AMY1C | [00278](http://www.ncbi.nlm.nih.gov/entrez/query.fcgi?db=Nucleotide&cmd=search&term=A_23_P23611) | amylase, alpha 1C; salivary | 3,16 |
| ANKRD15 | [23189](http://www.ncbi.nlm.nih.gov/entrez/query.fcgi?db=Nucleotide&cmd=search&term=A_23_P500130) | ankyrin repeat domain 15 | 4,51 |
| ARHGEF17 | [09828](http://www.ncbi.nlm.nih.gov/entrez/query.fcgi?db=Nucleotide&cmd=search&term=A_23_P76015) | Rho guanine nucleotide exchange factor (GEF) 17 | 2,81 |
| ATP10D | [57205](http://www.ncbi.nlm.nih.gov/entrez/query.fcgi?db=Nucleotide&cmd=search&term=A_23_P170280) | ATPase, Class V, type 10D | 2,18 |
| ATP9A | [10079](http://www.ncbi.nlm.nih.gov/entrez/query.fcgi?db=Nucleotide&cmd=search&term=A_23_P380614) | ATPase, Class II, type 9A | 2,68 |
| B3GALT4 | [08705](http://www.ncbi.nlm.nih.gov/entrez/query.fcgi?db=Nucleotide&cmd=search&term=A_23_P422071) | UDP-Gal:betaGlcNAc beta 1,3-galactosyltransferase, polypeptide 4 | 6,71 |
| BFSP1 | [00631](http://www.ncbi.nlm.nih.gov/entrez/query.fcgi?db=Nucleotide&cmd=search&term=A_23_P109171) | beaded filament structural protein 1, filensin | 5,53 |
| BZRAP1 | [09256](http://www.ncbi.nlm.nih.gov/entrez/query.fcgi?db=Nucleotide&cmd=search&term=A_24_P113131) | benzodiazapine receptor (peripheral) associated protein 1 | 3,83 |
| C16orf45 | [89927](http://www.ncbi.nlm.nih.gov/entrez/query.fcgi?db=Nucleotide&cmd=search&term=A_23_P326319) | chromosome 16 open reading frame 45 | 5,66 |
| C1orf170 | [84808](http://www.ncbi.nlm.nih.gov/entrez/query.fcgi?db=Nucleotide&cmd=search&term=A_23_P135742) | chromosome 1 open reading frame 170 | 2,66 |
| C1QTNF3 | [114899](http://www.ncbi.nlm.nih.gov/entrez/query.fcgi?db=Nucleotide&cmd=search&term=A_23_P122068) | C1q and tumor necrosis factor related protein 3 | 5,20 |
| C1R | [00715](http://www.ncbi.nlm.nih.gov/entrez/query.fcgi?db=Nucleotide&cmd=search&term=A_23_P125423) | complement component 1, r subcomponent | 10,05 |
| C1S | [00716](http://www.ncbi.nlm.nih.gov/entrez/query.fcgi?db=Nucleotide&cmd=search&term=A_23_P2492) | complement component 1, s subcomponent | 2,93 |
| CCDC48 | [79825](http://www.ncbi.nlm.nih.gov/entrez/query.fcgi?db=Nucleotide&cmd=search&term=A_23_P166566) | coiled-coil domain containing 48 | 3,22 |
| CIRBP | [01153](http://www.ncbi.nlm.nih.gov/entrez/query.fcgi?db=Nucleotide&cmd=search&term=A_23_P413456) | cold inducible RNA binding protein | 3,90 |
| CLSTN3 | [09746](http://www.ncbi.nlm.nih.gov/entrez/query.fcgi?db=Nucleotide&cmd=search&term=A_23_P53724) | calsyntenin 3 | 3,10 |
| CLU | [01191](http://www.ncbi.nlm.nih.gov/entrez/query.fcgi?db=Nucleotide&cmd=search&term=A_23_P215913) | clusterin | 3,11 |
| CLUL1 | [27098](http://www.ncbi.nlm.nih.gov/entrez/query.fcgi?db=Nucleotide&cmd=search&term=A_23_P55572) | clusterin-like 1 (retinal) | 5,17 |
| CMTM3 | 123920 | CKLF-like MARVEL transmembrane domain containing 3 | 2,48 |
| CNIH3 | [149111](http://www.ncbi.nlm.nih.gov/entrez/query.fcgi?db=Nucleotide&cmd=search&term=A_23_P384044) | cornichon homolog 3 (Drosophila) | 3,61 |
| COL27A1 | [85301](http://www.ncbi.nlm.nih.gov/entrez/query.fcgi?db=Nucleotide&cmd=search&term=A_23_P158096) | collagen, type XXVII, alpha 1 | 4,36 |
| COL5A2 | [01290](http://www.ncbi.nlm.nih.gov/entrez/query.fcgi?db=Nucleotide&cmd=search&term=A_23_P33196) | collagen, type V, alpha 2 | 4,68 |
| COMP | [01311](http://www.ncbi.nlm.nih.gov/entrez/query.fcgi?db=Nucleotide&cmd=search&term=A_24_P264943) | cartilage oligomeric matrix protein | 3,57 |
| CPE | [01363](http://www.ncbi.nlm.nih.gov/entrez/query.fcgi?db=Nucleotide&cmd=search&term=A_23_P259442) | carboxypeptidase E | 3,97 |
| CPLX1 | [10815](http://www.ncbi.nlm.nih.gov/entrez/query.fcgi?db=Nucleotide&cmd=search&term=A_24_P51909) | complexin 1 | 3,09 |
| CPQ | [10404](http://www.ncbi.nlm.nih.gov/entrez/query.fcgi?db=Nucleotide&cmd=search&term=A_23_P256735) | carboxypeptidase Q | 3,38 |
| CYBASC3 | [220002](http://www.ncbi.nlm.nih.gov/entrez/query.fcgi?db=Nucleotide&cmd=search&term=A_23_P342131) | cytochrome b, ascorbate dependent 3 | 8,54 |
| DHRS13 | [147015](http://www.ncbi.nlm.nih.gov/entrez/query.fcgi?db=Nucleotide&cmd=search&term=A_23_P66719) | dehydrogenase/reductase (SDR family) member 13 | 4,05 |
| DMGDH | [29958](http://www.ncbi.nlm.nih.gov/entrez/query.fcgi?db=Nucleotide&cmd=search&term=A_23_P257803) | dimethylglycine dehydrogenase | 3,27 |
| DMRTC1 | [63947](http://www.ncbi.nlm.nih.gov/entrez/query.fcgi?db=Nucleotide&cmd=search&term=A_32_P179998) | DMRT-like family C1 | 4,14 |
| DNAJB2 | [03300](http://www.ncbi.nlm.nih.gov/entrez/query.fcgi?db=Nucleotide&cmd=search&term=A_24_P102981) | DnaJ (Hsp40) homolog, subfamily B, member 2 | 2,92 |
| DNAJC12 | [56521](http://www.ncbi.nlm.nih.gov/entrez/query.fcgi?db=Nucleotide&cmd=search&term=A_23_P127220) | DnaJ (Hsp40) homolog, subfamily C, member 12 | 3,46 |
| DTX3 | [196403](http://www.ncbi.nlm.nih.gov/entrez/query.fcgi?db=Nucleotide&cmd=search&term=A_23_P53247) | deltex 3 homolog (Drosophila) | 5,46 |
| ENO2 | [02026](http://www.ncbi.nlm.nih.gov/entrez/query.fcgi?db=Nucleotide&cmd=search&term=A_24_P236091) | enolase 2 (gamma, neuronal) | 3,95 |
| ENPP4 | [22875](http://www.ncbi.nlm.nih.gov/entrez/query.fcgi?db=Nucleotide&cmd=search&term=A_23_P70318) | ectonucleotide pyrophosphatase/phosphodiesterase 4 (putative function) | 3,04 |
| ENPP5 | [59084](http://www.ncbi.nlm.nih.gov/entrez/query.fcgi?db=Nucleotide&cmd=search&term=A_23_P214244) | ectonucleotide pyrophosphatase/phosphodiesterase 5 (putative function) | 4,06 |
| ETNK2 | [55224](http://www.ncbi.nlm.nih.gov/entrez/query.fcgi?db=Nucleotide&cmd=search&term=A_23_P46025) | ethanolamine kinase 2 | 2,82 |
| EXD3 | [54932](http://www.ncbi.nlm.nih.gov/entrez/query.fcgi?db=Nucleotide&cmd=search&term=A_24_P49183) | exonuclease 3'-5' domain containing 3 | 4,41 |
| FAM135A | [57579](http://www.ncbi.nlm.nih.gov/entrez/query.fcgi?db=Nucleotide&cmd=search&term=A_23_P398172) | family with sequence similarity 135, member A | 3,55 |
| FAM182B | [728882](http://www.ncbi.nlm.nih.gov/entrez/query.fcgi?db=Nucleotide&cmd=search&term=A_23_P166147) | family with sequence similarity 182, member B | 4,31 |
| FAM198B | [51313](http://www.ncbi.nlm.nih.gov/entrez/query.fcgi?db=Nucleotide&cmd=search&term=A_23_P218928) | family with sequence similarity 198, member B | 3,28 |
| FMO4 | [02329](http://www.ncbi.nlm.nih.gov/entrez/query.fcgi?db=Nucleotide&cmd=search&term=A_23_P160992) | flavin containing monooxygenase 4 | 2,58 |
| GAB3 | [139716](http://www.ncbi.nlm.nih.gov/entrez/query.fcgi?db=Nucleotide&cmd=search&term=A_23_P255126) | GRB2-associated binding protein 3 | 3,99 |
| GARNL3 | [84253](http://www.ncbi.nlm.nih.gov/entrez/query.fcgi?db=Nucleotide&cmd=search&term=A_24_P158314) | GTPase activating Rap/RanGAP domain-like 3 | 4,30 |
| GOLPH3L | [55204](http://www.ncbi.nlm.nih.gov/entrez/query.fcgi?db=Nucleotide&cmd=search&term=A_24_P345377) | golgi phosphoprotein 3-like | 2,80 |
| GPA33 | [10223](http://www.ncbi.nlm.nih.gov/entrez/query.fcgi?db=Nucleotide&cmd=search&term=A_24_P319374) | glycoprotein A33 (transmembrane) | 3,42 |
| GRAMD4 | [23151](http://www.ncbi.nlm.nih.gov/entrez/query.fcgi?db=Nucleotide&cmd=search&term=A_24_P23258) | GRAM domain containing 4 | 3,10 |
| GRB10 | [02887](http://www.ncbi.nlm.nih.gov/entrez/query.fcgi?db=Nucleotide&cmd=search&term=A_24_P235266) | growth factor receptor-bound protein 10 | 4,12 |
| GRIK1 | [02897](http://www.ncbi.nlm.nih.gov/entrez/query.fcgi?db=Nucleotide&cmd=search&term=A_23_P109286) | glutamate receptor, ionotropic, kainate 1 | 13,86 |
| GSTM3 | [02947](http://www.ncbi.nlm.nih.gov/entrez/query.fcgi?db=Nucleotide&cmd=search&term=A_24_P920534) | glutathione S-transferase M3 (brain) | 3,98 |
| HS6ST2 | [90161](http://www.ncbi.nlm.nih.gov/entrez/query.fcgi?db=Nucleotide&cmd=search&term=A_24_P105191) | heparan sulfate 6-O-sulfotransferase 2 | 3,38 |
| IL1B | [03553](http://www.ncbi.nlm.nih.gov/entrez/query.fcgi?db=Nucleotide&cmd=search&term=A_23_P79518) | interleukin 1, beta | 13,03 |
| IRX3 | [79191](http://www.ncbi.nlm.nih.gov/entrez/query.fcgi?db=Nucleotide&cmd=search&term=A_23_P152235) | iroquois homeobox protein 3 | 3,40 |
| KIAA1407 | [57577](http://www.ncbi.nlm.nih.gov/entrez/query.fcgi?db=Nucleotide&cmd=search&term=A_23_P419213) | KIAA1407 | 3,29 |
| KIAA1683 | [80726](http://www.ncbi.nlm.nih.gov/entrez/query.fcgi?db=Nucleotide&cmd=search&term=A_23_P130974) | KIAA1683 | 3,89 |
| LAMP2 | [03920](http://www.ncbi.nlm.nih.gov/entrez/query.fcgi?db=Nucleotide&cmd=search&term=A_24_P396231) | lysosomal-associated membrane protein 2 | 3,31 |
| LARP6 | [55323](http://www.ncbi.nlm.nih.gov/entrez/query.fcgi?db=Nucleotide&cmd=search&term=A_23_P117782) | La ribonucleoprotein domain family, member 6 | 4,68 |
| LOH11CR2A | [04013](http://www.ncbi.nlm.nih.gov/entrez/query.fcgi?db=Nucleotide&cmd=search&term=A_23_P98455) | loss of heterozygosity, 11, chromosomal region 2, gene A | 2,78 |
| LRRC6 | [23639](http://www.ncbi.nlm.nih.gov/entrez/query.fcgi?db=Nucleotide&cmd=search&term=A_23_P112004) | leucine rich repeat containing 6 | 2,65 |
| LZTS3 | [09762](http://www.ncbi.nlm.nih.gov/entrez/query.fcgi?db=Nucleotide&cmd=search&term=A_23_P380298) | leucine zipper, putative tumor suppressor family member 3 | 3,94 |
| MAN1C1 | [57134](http://www.ncbi.nlm.nih.gov/entrez/query.fcgi?db=Nucleotide&cmd=search&term=A_23_P103601) | mannosidase, alpha, class 1C, member 1 | 3,06 |
| MAPRE2 | [10982](http://www.ncbi.nlm.nih.gov/entrez/query.fcgi?db=Nucleotide&cmd=search&term=A_23_P89835) | microtubule-associated protein, RP/EB family, member 2 | 3,92 |
| MARCH1 | [55016](http://www.ncbi.nlm.nih.gov/entrez/query.fcgi?db=Nucleotide&cmd=search&term=A_23_P7262) | membrane-associated ring finger (C3HC4) 1 | 2,65 |
| MDGA1 | [266727](http://www.ncbi.nlm.nih.gov/entrez/query.fcgi?db=Nucleotide&cmd=search&term=A_23_P310460) | MAM domain containing glycosylphosphatidylinositol anchor 1 | 4,72 |
| MFSD4 | [148808](http://www.ncbi.nlm.nih.gov/entrez/query.fcgi?db=Nucleotide&cmd=search&term=A_24_P51115) | major facilitator superfamily domain containing 4 | 2,69 |
| MME | [04311](http://www.ncbi.nlm.nih.gov/entrez/query.fcgi?db=Nucleotide&cmd=search&term=A_24_P260101) | membrane metallo-endopeptidase | 3,61 |
| MORN5 | [254956](http://www.ncbi.nlm.nih.gov/entrez/query.fcgi?db=Nucleotide&cmd=search&term=A_24_P401491) | MORN repeat containing 5 | 2,57 |
| MPP1 | [04354](http://www.ncbi.nlm.nih.gov/entrez/query.fcgi?db=Nucleotide&cmd=search&term=A_23_P171296) | membrane protein, palmitoylated 1, 55kDa | 5,31 |
| MROH8 | [140699](http://www.ncbi.nlm.nih.gov/entrez/query.fcgi?db=Nucleotide&cmd=search&term=A_23_P421811) | maestro heat-like repeat family member 8 | 2,67 |
| MSRB3 | [253827](http://www.ncbi.nlm.nih.gov/entrez/query.fcgi?db=Nucleotide&cmd=search&term=A_24_P273726) | methionine sulfoxide reductase B3 | 2,81 |
| MVP | [09961](http://www.ncbi.nlm.nih.gov/entrez/query.fcgi?db=Nucleotide&cmd=search&term=A_23_P88819) | major vault protein | 3,62 |
| N4BP2L2 | [10443](http://www.ncbi.nlm.nih.gov/entrez/query.fcgi?db=Nucleotide&cmd=search&term=A_23_P65262) | NEDD4 binding protein 2-like 2 | 3,19 |
| NAB1 | [04664](http://www.ncbi.nlm.nih.gov/entrez/query.fcgi?db=Nucleotide&cmd=search&term=A_24_P191417) | NGFI-A binding protein 1 (EGR1 binding protein 1) | 4,39 |
| NBEA | [26960](http://www.ncbi.nlm.nih.gov/entrez/query.fcgi?db=Nucleotide&cmd=search&term=A_23_P65278) | neurobeachin | 3,82 |
| NCAM2 | [04685](http://www.ncbi.nlm.nih.gov/entrez/query.fcgi?db=Nucleotide&cmd=search&term=A_32_P199429) | neural cell adhesion molecule 2 | 3,18 |
| NEFL | [04747](http://www.ncbi.nlm.nih.gov/entrez/query.fcgi?db=Nucleotide&cmd=search&term=A_24_P18137) | neurofilament, light polypeptide 68kDa | 2,74 |
| NELL2 | [04753](http://www.ncbi.nlm.nih.gov/entrez/query.fcgi?db=Nucleotide&cmd=search&term=A_23_P10025) | NEL-like 2 (chicken) | 2,88 |
| NFIL3 | [04783](http://www.ncbi.nlm.nih.gov/entrez/query.fcgi?db=Nucleotide&cmd=search&term=A_23_P32253) | nuclear factor, interleukin 3 regulated | 2,99 |
| NLGN1 | [22871](http://www.ncbi.nlm.nih.gov/entrez/query.fcgi?db=Nucleotide&cmd=search&term=A_23_P18123) | neuroligin 1 | 3,58 |
| NR3C2 | [04306](http://www.ncbi.nlm.nih.gov/entrez/query.fcgi?db=Nucleotide&cmd=search&term=A_23_P392470) | nuclear receptor subfamily 3, group C, member 2 | 2,88 |
| NSG1 | [27065](http://www.ncbi.nlm.nih.gov/entrez/query.fcgi?db=Nucleotide&cmd=search&term=A_23_P371107) | neuron specific gene family member 1 | 3,56 |
| NTF5 | [04909](http://www.ncbi.nlm.nih.gov/entrez/query.fcgi?db=Nucleotide&cmd=search&term=A_23_P4899) | neurotrophin 5 (neurotrophin 4/5) | 3,56 |
| OBSCN | [84033](http://www.ncbi.nlm.nih.gov/entrez/query.fcgi?db=Nucleotide&cmd=search&term=A_24_P119685) | obscurin, cytoskeletal calmodulin and titin-interacting RhoGEF | 3,26 |
| OBSL1 | [23363](http://www.ncbi.nlm.nih.gov/entrez/query.fcgi?db=Nucleotide&cmd=search&term=A_23_P335695) | obscurin-like 1 | 2,78 |
| OPRK1 | [04986](http://www.ncbi.nlm.nih.gov/entrez/query.fcgi?db=Nucleotide&cmd=search&term=A_32_P33576) | opioid receptor, kappa 1 | 9,90 |
| PA2G4 | [05036](http://www.ncbi.nlm.nih.gov/entrez/query.fcgi?db=Nucleotide&cmd=search&term=A_32_P224327) | proliferation-associated 2G4, 38kDa | 3,23 |
| PAM | [05066](http://www.ncbi.nlm.nih.gov/entrez/query.fcgi?db=Nucleotide&cmd=search&term=A_23_P213678) | peptidylglycine alpha-amidating monooxygenase | 2,95 |
| PCDH11Y | [83259](http://www.ncbi.nlm.nih.gov/entrez/query.fcgi?db=Nucleotide&cmd=search&term=A_32_P137980) | protocadherin 11 Y-linked | 12,30 |
| PCDHB14 | [56122](http://www.ncbi.nlm.nih.gov/entrez/query.fcgi?db=Nucleotide&cmd=search&term=A_23_P133236) | protocadherin beta 14 | 3,34 |
| PCDHGA8 | [09708](http://www.ncbi.nlm.nih.gov/entrez/query.fcgi?db=Nucleotide&cmd=search&term=A_23_P354734) | protocadherin gamma subfamily A, 8 | 2,81 |
| PDGFA | [05154](http://www.ncbi.nlm.nih.gov/entrez/query.fcgi?db=Nucleotide&cmd=search&term=A_23_P113701) | platelet-derived growth factor alpha polypeptide | 3,60 |
| PEBP4 | [157310](http://www.ncbi.nlm.nih.gov/entrez/query.fcgi?db=Nucleotide&cmd=search&term=A_23_P157636) | phosphatidylethanolamine-binding protein 4 | 3,81 |
| PHF1 | [05252](http://www.ncbi.nlm.nih.gov/entrez/query.fcgi?db=Nucleotide&cmd=search&term=A_23_P156732) | PHD finger protein 1 | 3,37 |
| PLA2G2A | [05320](http://www.ncbi.nlm.nih.gov/entrez/query.fcgi?db=Nucleotide&cmd=search&term=A_23_P321949) | phospholipase A2, group IIA (platelets, synovial fluid) | 9,56 |
| PLEKHB1 | [58473](http://www.ncbi.nlm.nih.gov/entrez/query.fcgi?db=Nucleotide&cmd=search&term=A_23_P53211) | pleckstrin homology domain containing, family B (evectins) member 1 | 3,74 |
| PNMA2 | [10687](http://www.ncbi.nlm.nih.gov/entrez/query.fcgi?db=Nucleotide&cmd=search&term=A_24_P389415) | paraneoplastic antigen MA2 | 5,60 |
| PPP2R5B | [05526](http://www.ncbi.nlm.nih.gov/entrez/query.fcgi?db=Nucleotide&cmd=search&term=A_23_P35796) | protein phosphatase 2, regulatory subunit B', beta isoform | 2,88 |
| PRMT2 | [03275](http://www.ncbi.nlm.nih.gov/entrez/query.fcgi?db=Nucleotide&cmd=search&term=A_23_P80156) | protein arginine methyltransferase 2 | 2,33 |
| PRSS16 | [10279](http://www.ncbi.nlm.nih.gov/entrez/query.fcgi?db=Nucleotide&cmd=search&term=A_23_P340131) | protease, serine, 16 (thymus) | 2,80 |
| PSMG3-AS1 | [114796](http://www.ncbi.nlm.nih.gov/entrez/query.fcgi?db=Nucleotide&cmd=search&term=A_23_P123086) | PSMG3 antisense RNA 1 (head to head) | 5,46 |
| PYROXD2 | [84795](http://www.ncbi.nlm.nih.gov/entrez/query.fcgi?db=Nucleotide&cmd=search&term=A_23_P24157) | pyridine nucleotide-disulphide oxidoreductase domain 2 | 3,19 |
| RGS11 | [08786](http://www.ncbi.nlm.nih.gov/entrez/query.fcgi?db=Nucleotide&cmd=search&term=A_23_P118122) | regulator of G-protein signalling 11 | 4,04 |
| RHCE | [06006](http://www.ncbi.nlm.nih.gov/entrez/query.fcgi?db=Nucleotide&cmd=search&term=A_23_P62634) | Rh blood group, CcEe antigens | 3,01 |
| RIMBP3 | [85376](http://www.ncbi.nlm.nih.gov/entrez/query.fcgi?db=Nucleotide&cmd=search&term=A_23_P154962) | RIMS binding protein 3 | 2,85 |
| ROR2 | [04920](http://www.ncbi.nlm.nih.gov/entrez/query.fcgi?db=Nucleotide&cmd=search&term=A_23_P158318) | receptor tyrosine kinase-like orphan receptor 2 | 3,74 |
| SERHL2 | [253190](http://www.ncbi.nlm.nih.gov/entrez/query.fcgi?db=Nucleotide&cmd=search&term=A_23_P120953) | serine hydrolase-like 2 | 3,03 |
| SESN1 | [27244](http://www.ncbi.nlm.nih.gov/entrez/query.fcgi?db=Nucleotide&cmd=search&term=A_23_P93562) | sestrin 1 | 2,31 |
| SEZ6L2 | [26470](http://www.ncbi.nlm.nih.gov/entrez/query.fcgi?db=Nucleotide&cmd=search&term=A_24_P116587) | seizure related 6 homolog (mouse)-like 2 | 2,98 |
| SH3BGRL | [06451](http://www.ncbi.nlm.nih.gov/entrez/query.fcgi?db=Nucleotide&cmd=search&term=A_23_P148297) | SH3 domain binding glutamic acid-rich protein like | 3,03 |
| SI | [06476](http://www.ncbi.nlm.nih.gov/entrez/query.fcgi?db=Nucleotide&cmd=search&term=A_32_P302205) | sucrase-isomaltase (alpha-glucosidase) | 6,40 |
| SLC12A2 | [06558](http://www.ncbi.nlm.nih.gov/entrez/query.fcgi?db=Nucleotide&cmd=search&term=A_32_P25437) | solute carrier family 12 (sodium/potassium/chloride transporters), member 2 | 3,24 |
| SLC26A11 | [284129](http://www.ncbi.nlm.nih.gov/entrez/query.fcgi?db=Nucleotide&cmd=search&term=A_23_P125078) | solute carrier family 26, member 11 | 2,96 |
| SLC35F3 | [148641](http://www.ncbi.nlm.nih.gov/entrez/query.fcgi?db=Nucleotide&cmd=search&term=A_23_P422212) | solute carrier family 35, member F3 | 3,31 |
| SLC9B1 | [150159](http://www.ncbi.nlm.nih.gov/entrez/query.fcgi?db=Nucleotide&cmd=search&term=A_23_P415611) | solute carrier family 9, subfamily B (NHA1, cation proton antiporter 1), member 1 | 3,58 |
| SNRK | [54861](http://www.ncbi.nlm.nih.gov/entrez/query.fcgi?db=Nucleotide&cmd=search&term=A_23_P211985) | SNF related kinase | 2,95 |
| SORL1 | [06653](http://www.ncbi.nlm.nih.gov/entrez/query.fcgi?db=Nucleotide&cmd=search&term=A_23_P87049) | sortilin-related receptor, L(DLR class) A repeats-containing | 3,60 |
| SPINK2 | [06691](http://www.ncbi.nlm.nih.gov/entrez/query.fcgi?db=Nucleotide&cmd=search&term=A_23_P155688) | serine peptidase inhibitor, Kazal type 2 (acrosin-trypsin inhibitor) | 8,95 |
| SRGAP3 | [09901](http://www.ncbi.nlm.nih.gov/entrez/query.fcgi?db=Nucleotide&cmd=search&term=A_23_P358410) | SLIT-ROBO Rho GTPase activating protein 3 | 3,08 |
| ST5 | [06764](http://www.ncbi.nlm.nih.gov/entrez/query.fcgi?db=Nucleotide&cmd=search&term=A_23_P24884) | suppression of tumorigenicity 5 | 2,51 |
| ST6GALNAC5 | [81849](http://www.ncbi.nlm.nih.gov/entrez/query.fcgi?db=Nucleotide&cmd=search&term=A_23_P33093) | ST6 (alpha-N-acetyl-neuraminyl-2,3-beta-galactosyl-1,3)-N-acetylgalactosaminide alpha-2,6-sialyltransferase 5 | 2,95 |
| ST7 | [07982](http://www.ncbi.nlm.nih.gov/entrez/query.fcgi?db=Nucleotide&cmd=search&term=A_24_P141019) | suppression of tumorigenicity 7 | 2,79 |
| STBD1 | [08987](http://www.ncbi.nlm.nih.gov/entrez/query.fcgi?db=Nucleotide&cmd=search&term=A_32_P83811) | starch binding domain 1 | 2,77 |
| STMN3 | [50861](http://www.ncbi.nlm.nih.gov/entrez/query.fcgi?db=Nucleotide&cmd=search&term=A_23_P165927) | stathmin-like 3 | 2,95 |
| SUSD2 | [56241](http://www.ncbi.nlm.nih.gov/entrez/query.fcgi?db=Nucleotide&cmd=search&term=A_23_P314101) | sushi domain containing 2 | 5,00 |
| SUSD4 | [55061](http://www.ncbi.nlm.nih.gov/entrez/query.fcgi?db=Nucleotide&cmd=search&term=A_24_P259607) | sushi domain containing 4 | 3,86 |
| SUSD6 | [09766](http://www.ncbi.nlm.nih.gov/entrez/query.fcgi?db=Nucleotide&cmd=search&term=A_23_P371787) | sushi domain containing 6 | 3,10 |
| TECPR2 | [09895](http://www.ncbi.nlm.nih.gov/entrez/query.fcgi?db=Nucleotide&cmd=search&term=A_23_P394216) | tectonin beta-propeller repeat containing 2 | 2,45 |
| THBS4 | [07060](http://www.ncbi.nlm.nih.gov/entrez/query.fcgi?db=Nucleotide&cmd=search&term=A_24_P260443) | thrombospondin 4 | 5,42 |
| THRA | [07067](http://www.ncbi.nlm.nih.gov/entrez/query.fcgi?db=Nucleotide&cmd=search&term=A_24_P262407) | thyroid hormone receptor, alpha (erythroblastic leukemia viral (v-erb-a) oncogene homolog, avian) | 2,92 |
| TLL2 | [07093](http://www.ncbi.nlm.nih.gov/entrez/query.fcgi?db=Nucleotide&cmd=search&term=A_23_P404778) | tolloid-like 2 | 6,72 |
| TMCO3 | [55002](http://www.ncbi.nlm.nih.gov/entrez/query.fcgi?db=Nucleotide&cmd=search&term=A_23_P205188) | transmembrane and coiled-coil domains 3 | 2,87 |
| TMEM106B | [54664](http://www.ncbi.nlm.nih.gov/entrez/query.fcgi?db=Nucleotide&cmd=search&term=A_32_P353072) | transmembrane protein 106B | 2,62 |
| TMEM170 | [124491](http://www.ncbi.nlm.nih.gov/entrez/query.fcgi?db=Nucleotide&cmd=search&term=A_24_P504050) | transmembrane protein 170 | 3,25 |
| TMEM38B | [55151](http://www.ncbi.nlm.nih.gov/entrez/query.fcgi?db=Nucleotide&cmd=search&term=A_23_P60259) | transmembrane protein 38B | 5,62 |
| TMEM59L | [25789](http://www.ncbi.nlm.nih.gov/entrez/query.fcgi?db=Nucleotide&cmd=search&term=A_23_P309155) | transmembrane protein 59-like | 4,87 |
| TNFRSF12A | [51330](http://www.ncbi.nlm.nih.gov/entrez/query.fcgi?db=Nucleotide&cmd=search&term=A_23_P49338) | tumor necrosis factor receptor superfamily, member 12A | 2,77 |
| TNFSF4 | [07292](http://www.ncbi.nlm.nih.gov/entrez/query.fcgi?db=Nucleotide&cmd=search&term=A_23_P126836) | tumor necrosis factor (ligand) superfamily, member 4 (tax-transcriptionally activated glycoprotein 1, 34kDa) | 3,21 |
| TRIQK | [286144](http://www.ncbi.nlm.nih.gov/entrez/query.fcgi?db=Nucleotide&cmd=search&term=A_32_P117313) | triple QxxK/R motif containing | 2,54 |
| TRPC1 | [07220](http://www.ncbi.nlm.nih.gov/entrez/query.fcgi?db=Nucleotide&cmd=search&term=A_24_P28977) | transient receptor potential cation channel, subfamily C, member 1 | 3,33 |
| TSGA10 | [80705](http://www.ncbi.nlm.nih.gov/entrez/query.fcgi?db=Nucleotide&cmd=search&term=A_23_P17103) | testis specific, 10 | 2,92 |
| TSHZ3 | [57616](http://www.ncbi.nlm.nih.gov/entrez/query.fcgi?db=Nucleotide&cmd=search&term=A_23_P361014) | teashirt family zinc finger 3 | 4,64 |
| TSLP | [85480](http://www.ncbi.nlm.nih.gov/entrez/query.fcgi?db=Nucleotide&cmd=search&term=A_23_P121987) | thymic stromal lymphopoietin | 3,31 |
| TTC18 | [118491](http://www.ncbi.nlm.nih.gov/entrez/query.fcgi?db=Nucleotide&cmd=search&term=A_23_P326931) | tetratricopeptide repeat domain 18 | 4,16 |
| TXNIP | [10628](http://www.ncbi.nlm.nih.gov/entrez/query.fcgi?db=Nucleotide&cmd=search&term=A_23_P97700) | thioredoxin interacting protein | 4,16 |
| TYRP1 | [07306](http://www.ncbi.nlm.nih.gov/entrez/query.fcgi?db=Nucleotide&cmd=search&term=A_23_P94403) | tyrosinase-related protein 1 | 5,25 |
| USP11 | [08237](http://www.ncbi.nlm.nih.gov/entrez/query.fcgi?db=Nucleotide&cmd=search&term=A_23_P171366) | ubiquitin specific peptidase 11 | 4,91 |
| VIM | [07431](http://www.ncbi.nlm.nih.gov/entrez/query.fcgi?db=Nucleotide&cmd=search&term=A_23_P161194) | vimentin | 6,03 |
| WDR66 | [144406](http://www.ncbi.nlm.nih.gov/entrez/query.fcgi?db=Nucleotide&cmd=search&term=A_23_P363275) | WD repeat domain 66 | 3,05 |
| ZBTB4 | [57659](http://www.ncbi.nlm.nih.gov/entrez/query.fcgi?db=Nucleotide&cmd=search&term=A_23_P100654) | zinc finger and BTB domain containing 4 | 2,71 |
| ZMAT1 | [84460](http://www.ncbi.nlm.nih.gov/entrez/query.fcgi?db=Nucleotide&cmd=search&term=A_24_P11100) | zinc finger, matrin type 1 | 4,62 |
| ZNF177 | [07730](http://www.ncbi.nlm.nih.gov/entrez/query.fcgi?db=Nucleotide&cmd=search&term=A_23_P164706) | zinc finger protein 177 | 2,48 |
| FC*: fold change | |  |  |
