# Supplementary material for: Up-Regulated Expression of LAMP2 and Autophagy Activity during Neuroendocrine Differentiation of Prostate Cancer LNCaP Cells
Source: PLoS One. 2016 Sep 14;11(9):e0162977. doi: 10.1371/journal.pone.0162977 (PMC5023108; doi:10.1371/journal.pone.0162977)
Supplement: S2 Table — Down-regulated genes. (DOCX) [file pone.0162977.s005.docx]

**Table S2: DEGs in NE cells. Down-regulated genes**

Genes down-regulated in neuroendocrine differentiated LNCaP cells induced by serum deprivation

| **SYMBOL** | **GENE ID** | **GENE NAME** | **FC*** |
| --- | --- | --- | --- |
| AADAT | 51166 | aminoadipate aminotransferase | - 6,94 |
| ADAM7 | 8756 | ADAM metallopeptidase domain 7 | - 2,26 |
| AIM1 | 202 | absent in melanoma 1 | - 4,01 |
| ANLN | 54443 | anillin, actin binding protein | - 16,42 |
| ARHGAP11A | 9824 | Rho GTPase activating protein 11A | - 14,41 |
| ASF1B | 55723 | ASF1 anti-silencing function 1 homolog B (S. cerevisiae) | - 18,44 |
| ASPM | 259266 | asp (abnormal spindle) homolog, microcephaly associated (Drosophila) | - 34,95 |
| ATAD2 | 29028 | ATPase family, AAA domain containing 2 | - 8,05 |
| ATAD5 | 79915 | ATPase family, AAA domain containing 5 | - 2,68 |
| ATPBD4 | 89978 | ATP binding domain 4 | - 2,24 |
| AURKA | 6790 | aurora kinase A | - 20,51 |
| AURKB | 9212 | aurora kinase B | - 10,11 |
| BCL2L12 | 83596 | BCL2-like 12 (proline rich) | - 3,13 |
| BCL2L13 | 23786 | BCL2-like 13 (apoptosis facilitator) | - 2,66 |
| BDKRB1 | 623 | bradykinin receptor B1 | - 3,87 |
| BIRC5 | 332 | baculoviral IAP repeat-containing 5 (survivin) | - 80,84 |
| BLM | 641 | Bloom syndrome | - 12,56 |
| BRCA1 | 672 | breast cancer 1, early onset | - 12,34 |
| BUB1 | 699 | BUB1 budding uninhibited by benzimidazoles 1 homolog (yeast) | - 19,36 |
| BUB3 | 9184 | BUB3 budding uninhibited by benzimidazoles 3 homolog (yeast) | - 3,36 |
| BZW2 | 28969 | basic leucine zipper and W2 domains 2 | - 2,66 |
| C12orf48 | 55010 | chromosome 12 open reading frame 48 | - 5,11 |
| C13orf3 | 221150 | chromosome 13 open reading frame 3 | - 24,03 |
| C15orf42 | 90381 | chromosome 15 open reading frame 42 | - 30,72 |
| C16orf59 | 80178 | chromosome 16 open reading frame 59 | - 4,18 |
| C16orf75 | 116028 | chromosome 16 open reading frame 75 | - 7,61 |
| C18orf56 | 494514 | chromosome 18 open reading frame 56 | - 5,08 |
| C1orf135 | 79000 | chromosome 1 open reading frame 135 | - 7,03 |
| C6orf173 | 387103 | chromosome 6 open reading frame 173 | - 13,54 |
| C9orf100 | 84904 | chromosome 9 open reading frame 100 | - 6,56 |
| C9orf140 | 89958 | chromosome 9 open reading frame 140 | - 4,73 |
| CCDC86 | 79080 | coiled-coil domain containing 86 | - 2,28 |
| CCNA2 | 890 | cyclin A2 | - 23,64 |
| CCNB1 | 891 | cyclin B1 | - 13,45 |
| CCNB2 | 9133 | cyclin B2 | - 46,49 |
| CDC2 | 983 | cell division cycle 2, G1 to S and G2 to M | - 17,67 |
| CDC20 | 991 | cell division cycle 20 homolog (S. cerevisiae) | - 22,83 |
| CDC25A | 993 | cell division cycle 25 homolog A (S. pombe) | - 6,53 |
| CDC25C | 995 | cell division cycle 25 homolog C (S. pombe) | - 16,43 |
| CDC45L | 8318 | CDC45 cell division cycle 45-like (S. cerevisiae) | - 51,34 |
| CDC6 | 990 | cell division cycle 6 homolog (S. cerevisiae) | - 28,19 |
| CDCA2 | 157313 | cell division cycle associated 2 | - 32,83 |
| CDCA3 | 83461 | cell division cycle associated 3 | - 16,70 |
| CDCA5 | 113130 | cell division cycle associated 5 | - 19,75 |
| CDCA7 | 83879 | cell division cycle associated 7 | - 4,55 |
| CDCA8 | 55143 | cell division cycle associated 8 | - 18,29 |
| CDKN3 | 1033 | cyclin-dependent kinase inhibitor 3 (CDK2-associated dual specificity phosphatase) | - 19,05 |
| CDT1 | 81620 | chromatin licensing and DNA replication factor 1 | - 7,20 |
| CENPA | 1058 | centromere protein A | - 25,74 |
| CENPE | 1062 | centromere protein E, 312kDa | - 15,75 |
| CENPF | 1063 | centromere protein F, 350/400ka (mitosin) | - 30,82 |
| CENPH | 64946 | centromere protein H | - 6,13 |
| CENPM | 79019 | centromere protein M | - 20,81 |
| CENPN | 55839 | centromere protein N | - 5,13 |
| CEP27 | 55142 | centrosomal protein 27kDa | - 2,75 |
| CEP55 | 55165 | centrosomal protein 55kDa | - 43,96 |
| CHAF1A | 10036 | chromatin assembly factor 1, subunit A (p150) | - 4,02 |
| CHEK1 | 1111 | CHK1 checkpoint homolog (S. pombe) | - 8,93 |
| CIT | 11113 | citron (rho-interacting, serine/threonine kinase 21) | - 13,30 |
| CLSPN | 63967 | claspin homolog (Xenopus laevis) | - 2,59 |
| COQ3 | 51805 | coenzyme Q3 homolog, methyltransferase (S. cerevisiae) | - 2,51 |
| CTSL2 | 1515 | cathepsin L2 | - 5,07 |
| DBF4 | 10926 | DBF4 homolog (S. cerevisiae) | - 7,14 |
| DCC1 | 79075 | DNA replication and sister chromatid cohesion 1 | - 4,77 |
| DDIAS | 220042 | DNA damage-induced apoptosis suppressor | - 12,83 |
| DEPDC1B | 55789 | DEP domain containing 1B | - 25,94 |
| DHFR | 1719 | dihydrofolate reductase | - 4,02 |
| DHFRL1 | 200895 | dihydrofolate reductase-like 1 | - 2,85 |
| DHRS2 | 10202 | dehydrogenase/reductase (SDR family) member 2 | - 6,25 |
| DIAPH3 | 81624 | diaphanous homolog 3 (Drosophila) | - 8,42 |
| HJURP | 55355 | Holliday junction recognition protein | - 29,88 |
| DLAT | 1737 | dihydrolipoamide S-acetyltransferase (E2 component of pyruvate dehydrogenase complex) | - 3,11 |
| DLG7 | 9787 | discs, large homolog 7 (Drosophila) | - 55,41 |
| DTL | 51514 | denticleless homolog (Drosophila) | - 16,83 |
| DYNLL2 | 140735 | dynein, light chain, LC8-type 2 | - 3,03 |
| E2F1 | 1869 | E2F transcription factor 1 | - 20,46 |
| E2F2 | 1870 | E2F transcription factor 2 | - 34,68 |
| E2F7 | 144455 | E2F transcription factor 7 | - 15,38 |
| ECE2 | 9718 | endothelin converting enzyme 2 | - 2,74 |
| ECT2 | 1894 | epithelial cell transforming sequence 2 oncogene | - 4,71 |
| EIF2C2 | 27161 | eukaryotic translation initiation factor 2C, 2 | - 3,13 |
| EIF5A | 1984 | eukaryotic translation initiation factor 5A | - 3,18 |
| ELOVL7 | 79993 | ELOVL family member 7, elongation of long chain fatty acids (yeast) | - 2,71 |
| ESCO2 | 157570 | establishment of cohesion 1 homolog 2 (S. cerevisiae) | - 21,48 |
| ESPL1 | 9700 | extra spindle pole bodies homolog 1 (S. cerevisiae) | - 20,45 |
| EXO1 | 9156 | exonuclease 1 | - 24,97 |
| EZH2 | 2146 | enhancer of zeste homolog 2 (Drosophila) | - 3,91 |
| FAM111B | 374393 | family with sequence similarity 111, member B | - 5,52 |
| FAM33A | 348235 | family with sequence similarity 33, member A | - 3,30 |
| FAM54A | 113115 | family with sequence similarity 54, member A | - 20,85 |
| FAM64A | 54478 | family with sequence similarity 64, member A | - 46,59 |
| FAM72A | 653573 | family with sequence similarity 72, member A | - 11,13 |
| FAM83D | 81610 | family with sequence similarity 83, member D | - 50,35 |
| FANCD2 | 2177 | Fanconi anemia, complementation group D2 | - 3,81 |
| FBXO10 | 26267 | F-box protein 10 | - 2,52 |
| FKBP5 | 2289 | FK506 binding protein 5 | - 3,24 |
| DDIAS | 220042 | DNA damage-induced apoptosis suppressor | - 12,83 |
| FOXM1 | 2305 | forkhead box M1 | - 23,38 |
| FREQ | 23413 | frequenin homolog (Drosophila) | - 2,33 |
| GABPB2 | 2553 | GA binding protein transcription factor, beta subunit 2 | - 2,92 |
| GAL | 51083 | galanin | - 3,26 |
| GCLM | 2730 | glutamate-cysteine ligase, modifier subunit | - 3,14 |
| GGH | 8836 | gamma-glutamyl hydrolase (conjugase, folylpolygammaglutamyl hydrolase) | - 3,97 |
| GINS2 | 51659 | GINS complex subunit 2 (Psf2 homolog) | - 11,24 |
| GINS3 | 64785 | GINS complex subunit 3 (Psf3 homolog) | - 5,36 |
| GLRX2 | 51022 | glutaredoxin 2 | - 3,33 |
| GNB4 | 59345 | guanine nucleotide binding protein (G protein), beta polypeptide 4 | - 5,06 |
| GNPNAT1 | 64841 | glucosamine-phosphate N-acetyltransferase 1 | - 2,52 |
| GPSM2 | 29899 | G-protein signalling modulator 2 (AGS3-like, C. elegans) | - 8,13 |
| GRPEL1 | 80273 | GrpE-like 1, mitochondrial (E. coli) | - 2,25 |
| GSG2 | 83903 | germ cell associated 2 (haspin) | - 11,50 |
| GTSE1 | 51512 | G-2 and S-phase expressed 1 | - 15,49 |
| HELLS | 3070 | helicase, lymphoid-specific | - 6,51 |
| HERV-FRD | 405754 | endogenous retrovirus group FRD member 1 | - 3,14 |
| HIST2H2AC | 8338 | histone cluster 2, H2ac | - 3,11 |
| HJURP | 55355 | Holliday junction recognition protein | - 29,88 |
| HMGB2 | 3148 | high-mobility group box 2 | - 10,22 |
| HMMR | 3161 | hyaluronan-mediated motility receptor (RHAMM) | - 69,21 |
| HNRPA3P1 | 10151 | heterogeneous nuclear ribonucleoprotein A3 pseudogene 1 | - 2,67 |
| HOMER2 | 9455 | homer homolog 2 (Drosophila) | - 3,38 |
| HSPC111 | 51491 | NOP16 nucleolar protein | - 2,49 |
| IMPA2 | 3613 | inositol(myo)-1(or 4)-monophosphatase 2 | - 2,84 |
| INCENP | 3619 | inner centromere protein antigens 135/155kDa | - 6,75 |
| INSIG1 | 3638 | insulin induced gene 1 | - 4,85 |
| KIAA0101 | 9768 | KIAA0101 | - 40,39 |
| KIAA0179 | 23076 | KIAA0179 | - 3,02 |
| KIAA1794 | 55215 | KIAA1794 | - 5,39 |
| KIF11 | 3832 | kinesin family member 11 | - 9,65 |
| KIF15 | 56992 | kinesin family member 15 | - 17,08 |
| KIF18A | 81930 | kinesin family member 18A | - 7,47 |
| KIF20A | 10112 | kinesin family member 20A | - 34,11 |
| KIF23 | 9493 | kinesin family member 23 | - 11,86 |
| KIF2C | 11004 | kinesin family member 2C | - 23,43 |
| KIF4A | 24137 | kinesin family member 4A | - 17,55 |
| KIFC1 | 3833 | kinesin family member C1 | - 24,92 |
| KLHL7 | 55975 | kelch-like 7 (Drosophila) | - 3,77 |
| KNTC2 | 10403 | kinetochore associated 2 | - 58,69 |
| KPNA2 | 3838 | karyopherin alpha 2 (RAG cohort 1, importin alpha 1) | - 5,24 |
| KRT19 | 3880 | keratin 19 | - 3,36 |
| LDHA | 3939 | lactate dehydrogenase A | - 2,99 |
| LMNB1 | 4001 | lamin B1 | - 8,29 |
| LRG1 | 116844 | leucine-rich alpha-2-glycoprotein 1 | - 3,89 |
| MAD2L1 | 4085 | MAD2 mitotic arrest deficient-like 1 (yeast) | - 13,12 |
| MAGOHB | 55110 | mago homolog B, exon junction complex core component | - 2,78 |
| MASTL | 84930 | microtubule associated serine/threonine kinase-like | - 3,77 |
| MCCC2 | 64087 | methylcrotonoyl-Coenzyme A carboxylase 2 (beta) | - 2,86 |
| MCM10 | 55388 | MCM10 minichromosome maintenance deficient 10 (S. cerevisiae) | - 21,89 |
| MCM7 | 4176 | MCM7 minichromosome maintenance deficient 7 (S. cerevisiae) | - 4,96 |
| MCM8 | 84515 | MCM8 minichromosome maintenance deficient 8 (S. cerevisiae) | - 4,11 |
| MELK | 9833 | maternal embryonic leucine zipper kinase | - 22,66 |
| MLF1IP | 79682 | MLF1 interacting protein | - 23,15 |
| MND1 | 84057 | meiotic nuclear divisions 1 homolog (S. cerevisiae) | - 28,70 |
| MNS1 | 55329 | meiosis-specific nuclear structural 1 | - 8,10 |
| MPP6 | 51678 | membrane protein, palmitoylated 6 (MAGUK p55 subfamily member 6) | - 2,69 |
| MSMB | 4477 | microseminoprotein, beta- | - 4,23 |
| MTHFD1L | 25902 | methylenetetrahydrofolate dehydrogenase (NADP+ dependent) 1-like | - 3,00 |
| MYBL2 | 4605 | v-myb myeloblastosis viral oncogene homolog (avian)-like 2 | - 19,88 |
| NCAPG | 64151 | non-SMC condensin I complex, subunit G | - 87,18 |
| NCAPH | 23397 | non-SMC condensin I complex, subunit H | - 1,00 |
| NEK2 | 4751 | NIMA (never in mitosis gene a)-related kinase 2 | - 19,82 |
| NFKBIL2 | 4796 | nuclear factor of kappa light polypeptide gene enhancer in B-cells inhibitor-like 2 | - 4,83 |
| NMU | 10874 | neuromedin U | - 70,62 |
| NP | 4860 | nucleoside phosphorylase | - 3,26 |
| NRGN | 4900 | neurogranin (protein kinase C substrate, RC3) | - 3,77 |
| NUDT1 | 4521 | nudix (nucleoside diphosphate linked moiety X)-type motif 1 | - 3,57 |
| NUF2 | 83540 | NUF2, NDC80 kinetochore complex component, homolog (S. cerevisiae) | - 26,19 |
| NUSAP1 | 51203 | nucleolar and spindle associated protein 1 | - 25,02 |
| OAZ3 | 51686 | ornithine decarboxylase antizyme 3 | - 3,15 |
| OIP5 | 11339 | Opa interacting protein 5 | - 16,36 |
| ORC6L | 23594 | origin recognition complex, subunit 6 like (yeast) | - 20,89 |
| PBK | 55872 | PDZ binding kinase | - 71,66 |
| PCGF5 | 84333 | polycomb group ring finger 5 | - 2,50 |
| PCNA | 5111 | proliferating cell nuclear antigen | - 5,15 |
| PDSS1 | 23590 | prenyl (decaprenyl) diphosphate synthase, subunit 1 | - 4,13 |
| PGC | 5225 | progastricsin (pepsinogen C) | - 4,21 |
| PIF1 | 80119 | PIF1 5'-to-3' DNA helicase homolog (S. cerevisiae) | - 37,12 |
| PIGW | 284098 | phosphatidylinositol glycan anchor biosynthesis, class W | - 2,40 |
| PKMYT1 | 9088 | protein kinase, membrane associated tyrosine/threonine 1 | - 17,52 |
| PLEKHK1 | 219790 | pleckstrin homology domain containing, family K member 1 | - 7,66 |
| PLK1 | 5347 | polo-like kinase 1 (Drosophila) | - 37,07 |
| PMM2 | 5373 | phosphomannomutase 2 | - 3,10 |
| POLE2 | 5427 | polymerase (DNA directed), epsilon 2 (p59 subunit) | - 5,42 |
| POLE3 | 54107 | polymerase (DNA directed), epsilon 3 (p17 subunit) | - 2,58 |
| POP1 | 10940 | processing of precursor 1, ribonuclease P/MRP subunit (S. cerevisiae) | - 4,76 |
| PPIF | 10105 | peptidylprolyl isomerase F (cyclophilin F) | - 2,55 |
| PPIL5 | 122769 | peptidylprolyl isomerase (cyclophilin)-like 5 | - 3,47 |
| PRIM1 | 5557 | primase, polypeptide 1, 49kDa | - 6,22 |
| PRIM2A | 5558 | primase, polypeptide 2A, 58kDa | - 2,56 |
| PSMC3IP | 29893 | PSMC3 interacting protein | - 7,74 |
| PTMA | 5757 | prothymosin, alpha (gene sequence 28) | - 2,88 |
| PTTG1 | 9232 | pituitary tumor-transforming 1 | - 45,60 |
| PTTG2 | 10744 | pituitary tumor-transforming 2 | - 32,40 |
| PTTG3 | 26255 | pituitary tumor-transforming 3 | - 9,30 |
| RACGAP1 | 29127 | Rac GTPase activating protein 1 | - 9,58 |
| RAD51AP1 | 10635 | RAD51 associated protein 1 | - 8,96 |
| RAD54L | 8438 | RAD54-like (S. cerevisiae) | - 31,56 |
| RBM18 | 92400 | RNA binding motif protein 18 | - 2,46 |
| RC3H2 | 54542 | ring finger and CCCH-type zinc finger domains 2 | - 3,13 |
| RCC1 | 1104 | regulator of chromosome condensation 1 | - 3,48 |
| RECQL4 | 9401 | RecQ protein-like 4 | - 6,65 |
| RFC4 | 5984 | replication factor C (activator 1) 4, 37kDa | - 3,91 |
| RFC5 | 5985 | replication factor C (activator 1) 5, 36.5kDa | - 4,50 |
| RRM2 | 6241 | ribonucleotide reductase M2 polypeptide | - 50,63 |
| SCD | 6319 | stearoyl-CoA desaturase (delta-9-desaturase) | - 3,75 |
| SEC22C | 9117 | SEC22 vesicle trafficking protein homolog C (S. cerevisiae) | - 2,80 |
| SGOL1 | 151648 | shugoshin-like 1 (S. pombe) | - 12,48 |
| SHCBP1 | 79801 | SHC SH2-domain binding protein 1 | - 24,71 |
| SLBP | 7884 | stem-loop (histone) binding protein | - 2,48 |
| SLC16A14 | 151473 | solute carrier family 16, member 14 (monocarboxylic acid transporter 14) | - 2,75 |
| SLC25A33 | 84275 | solute carrier family 25, member 33 | - 3,39 |
| SLED1 | 643036 | proteoglycan 3 pseudogene | - 4,11 |
| SMC4 | 10051 | structural maintenance of chromosomes 4 | - 5,90 |
| SNRPB | 6628 | small nuclear ribonucleoprotein polypeptides B and B1 | - 2,74 |
| SNRPD1 | 6632 | small nuclear ribonucleoprotein D1 polypeptide 16kDa | - 2,27 |
| SNX5 | 27131 | sorting nexin 5 | - 3,89 |
| SPAG5 | 10615 | sperm associated antigen 5 | - 13,54 |
| SPBC24 | 147841 | spindle pole body component 24 homolog (S. cerevisiae) | - 14,70 |
| SPBC25 | 57405 | spindle pole body component 25 homolog (S. cerevisiae) | - 50,14 |
| SPCS3 | 60559 | signal peptidase complex subunit 3 homolog (S. cerevisiae) | - 2,55 |
| SSH1 | 54434 | slingshot homolog 1 (Drosophila) | - 3,18 |
| ST6GALNAC1 | 55808 | ST6 (alpha-N-acetyl-neuraminyl-2,3-beta-galactosyl-1,3)-N-acetylgalactosaminide alpha-2,6-sialyltransferase 1 | - 5,15 |
| TARP | 445347 | TCR gamma alternate reading frame protein | - 9,12 |
| TIMM8A | 1678 | translocase of inner mitochondrial membrane 8 homolog A (yeast) | - 2,89 |
| TK1 | 7083 | thymidine kinase 1, soluble | - 22,49 |
| TKT | 7086 | transketolase (Wernicke-Korsakoff syndrome) | - 2,06 |
| TM4SF1 | 4071 | transmembrane 4 L six family member 1 | - 15,42 |
| TMEM177 | 80775 | transmembrane protein 177 | - 2,67 |
| TMEM48 | 55706 | transmembrane protein 48 | - 5,63 |
| TMPO | 7112 | thymopoietin | - 3,99 |
| TMSL8 | 11013 | thymosin-like 8 | - 14,19 |
| TOP2A | 7153 | topoisomerase (DNA) II alpha 170kDa | - 37,07 |
| TPX2 | 22974 | TPX2, microtubule-associated, homolog (Xenopus laevis) | - 16,02 |
| TROAP | 10024 | trophinin associated protein (tastin) | - 21,16 |
| TTK | 7272 | TTK protein kinase | - 19,33 |
| TUBA1B | 10376 | tubulin, alpha 1b | - 3,99 |
| TYMS | 7298 | thymidylate synthetase | - 16,64 |
| UBE2C | 11065 | ubiquitin-conjugating enzyme E2C | - 72,15 |
| UBE2S | 27338 | ubiquitin-conjugating enzyme E2S | - 3,30 |
| UBE3C | 9690 | ubiquitin protein ligase E3C | - 2,82 |
| UCHL1 | 7345 | ubiquitin carboxyl-terminal esterase L1 (ubiquitin thiolesterase) | - 2,79 |
| UCK2 | 7371 | uridine-cytidine kinase 2 | - 3,56 |
| UHRF1 | 29128 | ubiquitin-like, containing PHD and RING finger domains, 1 | - 15,91 |
| WDR4 | 10785 | WD repeat domain 4 | - 2,63 |
| WDR51A | 25886 | WD repeat domain 51A | - 9,15 |
| XPO4 | 64328 | exportin 4 | - 2,28 |
| XRCC2 | 7516 | X-ray repair complementing defective repair in Chinese hamster cells 2 | - 3,09 |
| ZNF367 | 195828 | zinc finger protein 367 | - 4,14 |
| ZNF695 | 57116 | zinc finger protein 695 | - 5,65 |
| ZWILCH | 55055 | Zwilch, kinetochore associated, homolog (Drosophila) | - 5,75 |
| ZWINT | 11130 | ZW10 interactor | - 19,15 |
| FC*: fold change | |  |  |
