# Supplementary material for: Up-Regulated Expression of LAMP2 and Autophagy Activity during Neuroendocrine Differentiation of Prostate Cancer LNCaP Cells
Source: PLoS One. 2016 Sep 14;11(9):e0162977. doi: 10.1371/journal.pone.0162977 (PMC5023108; doi:10.1371/journal.pone.0162977)
Supplement: S3 Table — FC*: Fold change. (DOCX) [file pone.0162977.s006.docx]

**Table S3**

List of genes included in GO: BP "Anatomical_Structure_Development" and "Signal_Transduction" gene sets that are UP or DOWN-regulated in neuroendocrine cells.

| **SYMBOL** | **GENE ID** | **GENE NAME** | **FC*** |
| --- | --- | --- | --- |
| ALDH5A1 | [7915](http://www.ncbi.nlm.nih.gov/entrez/query.fcgi?db=gene&cmd=Retrieve&dopt=Graphics&list_uids=7915) | aldehyde dehydrogenase 5 family, member A1 (succinate-semialdehyde dehydrogenase) | 2,59 |
| COL5A2 | 1290 | collagen, type V, alpha 2 | 4,68 |
| COMP | 1311 | cartilage oligomeric matrix protein | 3,57 |
| GRB10 | 2887 | growth factor receptor-bound protein 10 | 4,12 |
| GRIK1 | 2897 | glutamate receptor, ionotropic, kainate 1 | 13,86 |
| GSTM3 | 2947 | glutathione S-transferase M3 (brain) | 5,42 |
| IL1B | 3553 | interleukin 1, beta | 13,03 |
| MAPRE2 | 10982 | microtubule-associated protein, RP/EB family, member 2 | 3,92 |
| MDGA1 | 266727 | MAM domain containing glycosylphosphatidylinositol anchor 1 | 4,72 |
| MPP1 | [4354](http://www.ncbi.nlm.nih.gov/entrez/query.fcgi?db=gene&cmd=Retrieve&dopt=Graphics&list_uids=4354) | membrane protein, palmitoylated 1, 55kDa | 5,31 |
| NLGN1 | 22871 | neuroligin 1 | 3,58 |
| NR3C2 | 4306 | nuclear receptor subfamily 3, group C, member 2 | 2,88 |
| OPRK1 | 4986 | opioid receptor, kappa 1 | 9,90 |
| PCDHB14 | 56122 | protocadherin beta 14 | 3,34 |
| PDGFA | 5154 | platelet-derived growth factor alpha polypeptide | 3,60 |
| PRMT2 | 3275 | protein arginine methyltransferase 2 | 2,33 |
| RGS11 | 8786 | regulator of G-protein signalling 11 | 4,04 |
| ROR2 | [4920](http://www.ncbi.nlm.nih.gov/entrez/query.fcgi?db=gene&cmd=Retrieve&dopt=Graphics&list_uids=4920) | receptor tyrosine kinase-like orphan receptor 2 | 3,74 |
| SNRK | 54861 | SNF related kinase | 2,95 |
| STMN3 | 50861 | stathmin-like 3 | 2,95 |
| THBS4 | 7060 | thrombospondin 4 | 4,68 |
| BRCA1 | 672 | breast cancer 1, early onset | - 12,34 |
| CCNA2 | 890 | cyclin A2 | - 23,64 |
| CHEK1 | 1111 | CHK1 checkpoint homolog (S. pombe) | - 8,93 |
| CIT | 11113 | citron (rho-interacting, serine/threonine kinase 21) | - 13,30 |
| ECT2 | 1894 | epithelial cell transforming sequence 2 oncogene | - 4,71 |
| GPSM2 | 29899 | G-protein signalling modulator 2 (AGS3-like, C. elegans) | - 8,13 |
| GSG2 | 83903 | germ cell associated 2 (haspin) | - 11,50 |
| HOMER2 | 9455 | homer homolog 2 (Drosophila) | - 3,38 |
| IMPA2 | 3613 | inositol(myo)-1(or 4)-monophosphatase 2 | - 2,84 |
| KRT19 | 3880 | keratin 19 | - 3,36 |
| NMU | 10874 | neuromedin U | - 70,62 |
| NRGN | 4900 | neurogranin (protein kinase C substrate, RC3) | - 3,77 |
| RACGAP1 | 29127 | Rac GTPase activating protein 1 | - 9,58 |
| SSH1 | 54434 | slingshot homolog 1 (Drosophila) | - 3,18 |
| TIMM8A | 1678 | translocase of inner mitochondrial membrane 8 homolog A (yeast) | - 2,89 |
| TOP2A | 7153 | topoisomerase (DNA) II alpha 170kDa | - 37,07 |

FC*: Fold change.
